# FEN1 Inhibition as a Potential Novel Targeted Therapy against Breast Cancer and the Prognostic Relevance of FEN1

**DOI:** 10.3390/ijms25042110

**Published:** 2024-02-09

**Authors:** Johanna Berfelde, Laura S. Hildebrand, Lukas Kuhlmann, Rainer Fietkau, Luitpold V. Distel

**Affiliations:** 1 Department of Radiation Oncology, Universitätsklinikum Erlangen, Friedrich-Alexander-Universität Erlangen-Nürnberg (FAU), 91054 Erlangen, Germany; 2 Comprehensive Cancer Center Erlangen-Europäische Metropolregion Nürnberg (CCC ER-EMN), 91054 Erlangen, Germany

**Keywords:** FEN1, FEN1 inhibition, FEN1-IN-4, breast cancer cell lines, targeted therapy, ionizing radiation, apoptosis, necrosis, TNBC, radiosensitivity

## Abstract

To improve breast cancer treatment and to enable new strategies for therapeutic resistance, therapeutic targets are constantly being studied. Potential targets are proteins of DNA repair and replication and genomic integrity, such as Flap Endonuclease 1 (FEN1). This study investigated the effects of FEN1 inhibitor FEN1-IN-4 in combination with ionizing radiation on cell death, clonogenic survival, the cell cycle, senescence, doubling time, DNA double-strand breaks and micronuclei in breast cancer cells, breast cells and healthy skin fibroblasts. Furthermore, the variation in the baseline FEN1 level and its influence on treatment prognosis was investigated. The cell lines show specific response patterns in the aspects studied and have heterogeneous baseline FEN1 levels. FEN1-IN-4 has cytotoxic, cytostatic and radiosensitizing effects, expressed through increasing cell death by apoptosis and necrosis, G2M share, senescence, double-strand breaks and a reduced survival fraction. Nevertheless, some cells are less affected by the cytotoxicity and fibroblasts show a rather limited response. In vivo, high FEN1 mRNA expression worsens the prognosis of breast cancer patients. Due to the increased expression in breast cancer tissue, FEN1 could represent a new tumor and prognosis marker and FEN1-IN-4 may serve as a new potent agent in personalized medicine and targeted breast cancer therapy.

## 1. Introduction

Breast cancer is the most frequent malignoma in women and one of the most frequent malignant diseases overall [1]. In 2020, approximately 2.3 million women worldwide were diagnosed with breast cancer and 685,000 died from it [2]. It is estimated that if current trends continue, there will be more than 3 million new diagnoses and 1 million deaths from breast cancer will occur in 2040 [2]. In 80 to 90% of cases, breast cancer occurs sporadically and non-hereditarily due to multifactorial genetic damage [3]. Breast cancer is a multifactorial and complicated disease that can be grouped according to molecular features, which has also made it possible to identify predictive and prognostic gene patterns [4,5,6]. Subgroups with aberrantly biological, genetic, and transcriptional patterns can be recognized and imagined as “molecular portraits” [5,7]. This is also reflected in the different response of breast cancer to (neo-)adjuvant and targeted therapy [5,6]. It is problematic that the heterogeneous subtypes of breast cancer respond differently to therapies and therefore do not have the same outcome [8]. This also emphasizes that the conventional clinical (such as histology, tumor size, affected lymph node, and age) and pathological (receptor status of human epidermal growth factor receptor 2 (HER2), estrogen, and progesterone) classifications do not do justice to the complexity of this disease and are therefore limited as factors in therapeutic decisions and prognosis [8,9]. The major molecular “intrinsic” subtypes of breast cancer tumors in literature are Basal-like, HER2 enriched, Luminal A, Luminal B, triple-negative (divided in Basal, Claudin-low, metaplastic breast cancer, and more), and normal breast-like [6,7,9,10,11,12,13]. In contrast, breast cancer cell lines show an additional subdivision into Basal A and Basal B, whereas Luminal is not differentiated, and HER2-amplified cells are distributed among Luminal and Basal A rather than grouping together in a cluster [5]. In summary, the nomenclature of breast cancer is very diverse in the literature.

A total of 15–20% of all invasive breast cancer cases are triple-negative breast cancer (TNBC), an aggressive subgroup that is hard to medicate due to inherent or developed chemoresistance [14]. Moreover, although the subtypes of TNBC are similar, their pathway modifications and gene expression are different [15]. New therapies with novel targets for TNBC are urgently needed [14,16].

Most of conventional and new targeted therapies are based on one of the Hallmarks of Cancer [17]. The Hallmarks of Cancer also include “Genome instability & mutation” and “Enabling replicative immortality” [18]. One protein that could be affected is Flap Endonuclease 1 (FEN1). FEN1 is a highly conserved, structure-specific, and multifunctional enzyme involved in many DNA metabolic pathways, and it preserves DNA integrity [19,20,21]. FEN1 is a small protein of approximately 42 kDa and interacts with approximately 20 distinct proteins and works in protein complexes [22]. By means of endonuclease activity of FEN1, single-stranded DNA flaps with 5′ terminus can be cut [19]. Furthermore, FEN1 has 5′-3′ exonuclease activity specifically for double-stranded DNA [19,23]. FEN1 can also exert gap endonuclease activity to clear blocked replication forks [24]. In particular, the 5′-3′ exonuclease activity and gap endonuclease activity of FEN1 can support DNA fragmentation during apoptosis [25]. FEN1 is necessary during DNA synthesis of the lagging strand for the maturation of Okazaki fragments [26]. Additionally, FEN1 conserves the stability of telomere and ribosomal DNA and increases genome stability [22,27].

Hence, cancer therapy, such as radiotherapy and chemotherapy, is based on the induction of DNA damage, e.g., double-strand break (DSB), and DNA repair mechanisms are of high interest [28]. DSB is one of the most serious forms of DNA damage [29]. FEN1 is involved in DSB repair and thus supports genetic stability [30,31]. Genome stability is preserved by DNA replication and repair, which require structure-specific endonucleases like FEN1 [31]. Base excision repair (BER) is a major and highly conserved pathway for DNA repair and thus also contributes to DNA integrity [32,33,34]. There are two pathways of BER, the long-patch BER and the single-nucleotide BER [22]. Long-patch BER requires FEN1 to generate a nick by cutting the flap of the single strand to enable ligation [22,32,34,35]. Furthermore, FEN1 is already being integrated into models of non-homologous end joining, homologous recombination, and microhomology-mediated end joining [30,36,37]. FEN1 promotes DNA repair through homologous recombination by removing heterologous sequences at the spot of DNA damage [36]. In contrast, overexpression of FEN1 promotes mutations and genomic instability, which are characteristics of cancer [38].

FEN1 is also of great importance in malignant diseases, and the following examples show the clinical relevance and consequences of FEN1 overexpression. The overexpression of FEN1 could be associated with poor survival and high grade and stage in epithelial ovarian carcinomas [39]. In cholangiocarcinoma, FEN1 was also overexpressed compared to normal tissue and patients with this overexpression had a reduced disease-free survival [40]. In addition, FEN1 was overexpressed in gastric cancer and FEN1 expression was associated with clinicopathological characteristics [41]. In lung cancer, FEN1 overexpression enhanced tumor progression, whereas after down-regulation or FEN1 inhibition, cells became more sensitive to cisplatin treatment [42]. Furthermore, cervical cancers also overexpressed FEN1, and FEN1 inhibition increased the sensitivity to ionizing radiation (IR) of cervical cancer [43].

**Figure 1:**
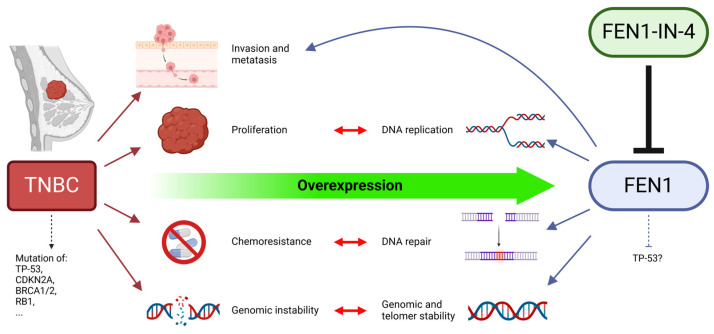
Flap Endonuclease 1 (FEN1) as a key gene and protein in the pathophysiology of triple-negative breast cancer (TNBC). Studies of hepatocellular carcinoma have led to suggestions that FEN1 inactivates tumor protein 53 (TP-53) [48]. Therefore, the dashed inhibitory arrow indicates a possible inhibition of FEN1 on TP-53. BRCA 1/2, breast cancer gene 1/2; CDKN2A, cyclin-dependent kinase inhibitor 2A; RB1, retinoblastoma gene 1. Adapted from [49]. Arrows imply an effect or property. Double arrows indicate an interaction. Created with BioRender.com.

There are various features of FEN1, some of them contradictory, that are of particular interest: the function of FEN1 at the cellular level, the varying expression of FEN1, the relevance of FEN1 for the prognosis of breast cancer, the search for potential structures for targeted therapy, and the effects of FEN1 inhibition. This study was also conducted explicitly on TNBC cell lines with the expectation of high and specific effects. The basic assumption was that certain characteristics of TNBC, such as chemoresistance and genomic instability, are closely related with features of FEN1 [14,44], as participating in DNA repair, DNA replication, and genomic stability and FEN1 overexpression has been implicated in the induction of chemoresistance (Figure 1) [22,45]. There is also an association between FEN1 and invasion and metastasis in TNBC [46]. In addition, FEN1 overexpression has already been detected in TNBC [46]. Since FEN1 is already suspected as a prognostic marker for ER+ cells and as a cause of insensitivity to tamoxifen, ER+ cells were examined [47]. Thus, the cell lines from the Luminal subtype were included. HER2+ cells account for another large proportion of breast cancer subtypes and were therefore selected for study. In this study, the effects of FEN1 inhibition with and without combined IR are studied, especially the influence of FEN1 expression on prognosis. The impact of FEN1-IN-4 is examined on eight breast cancer cells, breast epithelial cells, and healthy skin fibroblasts.

## 2. Results

### 2.1. The FEN1 Gene Is Overexpressed While Protein Levels Vary in Breast Cancer Cells

**Figure 2:**
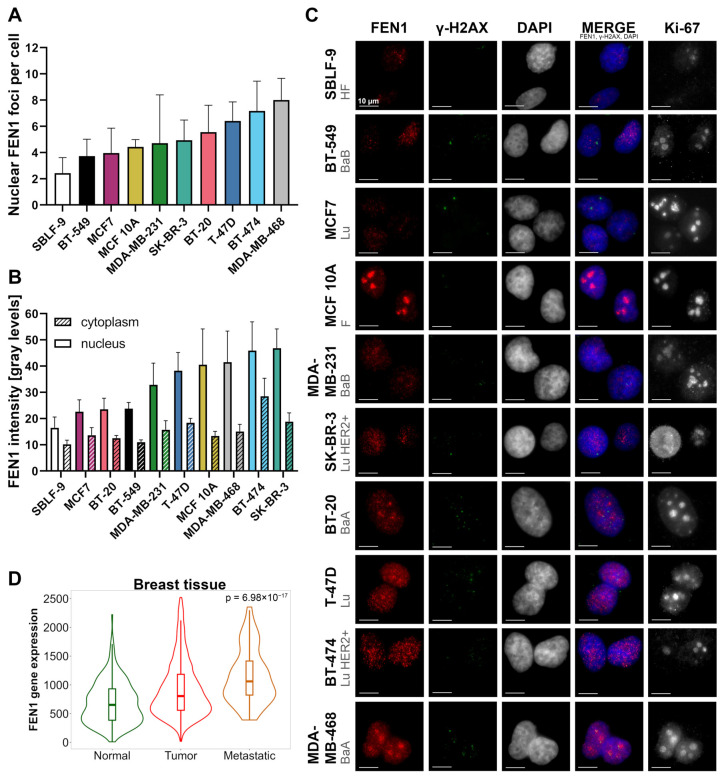
Analysis of the FEN1 base protein and gene expression level and visualization of the morphology of untreated cell lines and their respective FEN1 and γH2AX foci. The FEN1 evaluation was performed with standardized microscopy parameters and the same evaluation scheme. Color schemes of (**A**,**B**) are equivalent to facilitate comparison and both diagrams show the mean value of four independent experiments while error bars represent the standard deviation. (**A**) The cell lines are sorted according to ascending FEN1 foci level in the nucleus. Only foci that were clearly distinguishable from the background in both area and brightness were counted. (**B**) The cell lines are listed based on the increasing intensity of the FEN1 fluorescence signal in the nucleus. (**C**) The representative images show the distribution of FEN1 and γH2AX in relation to the nuclear morphology and proliferation activity. The rows represent the distinct cell lines sorted in descending order by increasing FEN1 foci base level. FEN1 foci (red), γH2AX foci (green), and DAPI (blue) are the first three columns and are also displayed as a merged image in column four next to the proliferation marker Ki-67 in column five. Scale bar represents 10 µm in all images. BaA, Basal A-like breast cancer cells; BaB, Basal B-like breast cancer cells; F, fibrocystic mammary epithelial cells; HF, healthy fibroblasts; Lu, Luminal-like breast cancer cells; Lu HER2+, Luminal HER2-amplified-like breast cancer cells. (**D**) Adapted from https://tnmplot.com (accessed on 2 December 2023). TNMplot data for the gene expression of the FEN1 gene in breast tissue (*p* = 6.98 × 10^−17^): breast cancer tumor (n = 7569), normal (n = 242) and metastatic tissue (n = 82). Significance was determined by the Kruskal–Wallis test.

Of great relevance for understanding the effects of an inhibitor was the investigation of whether the target protein FEN1 is evenly present and distributed in all cell lines and if the underlying FEN1 gene expression is increased in breast cancer. FEN1 levels were determined separately for nucleus and cytoplasm by immunostaining. FEN1 foci in the nucleus and FEN1 intensity in nucleus and cytoplasm were measured by standardized automated image analysis. Both the FEN1 intensity and the number of FEN1 foci were different between cell lines, but also within the same cell line (Figure 2A,B). The standardized count detected an average range of 2–8 foci and an average brightness score in nucleus of approximately 15 to 45 gray levels per cell. There was a positive correlation between the number of FEN1 foci and the intensity of FEN1 in the nucleus or cytoplasm as well as between the intensity of FEN1 in the cytoplasm and in the nucleus (Supplementary Figure S1). The accumulation of FEN1 in nucleoli regions in the cell lines T-47D, BT-20, MDA-MB-468, and MCF 10A, that can also be matched with Ki-67-positive areas (Figure 2C) was observed. FEN1 was localized in cytoplasm and nucleus in all cell lines. The baseline γH2AX foci count of untreated cells is displayed. The TNMplot database (https://tnmplot.com) (accessed on 2 December 2023) enabled a FEN1 gene expression analysis between normal, cancer, and metastatic breast tissue (Figure 2D) [50]. The median FEN1 gene expression was extended in tumor tissue and even more in metastatic tissue (*p* = 6.98 × 10^−17^). The pairwise comparison using Dunn’s test showed a significant difference for normal tissue and tumor (*p* = 1.22 × 10^−15^) or metastasis (*p* = 2.68 × 10^−12^) as well as between tumor and metastasis (*p* = 5.19 × 10^−4^).

### 2.2. FEN1-IN-4 Reduces the Survival Fraction

**Figure 3:**
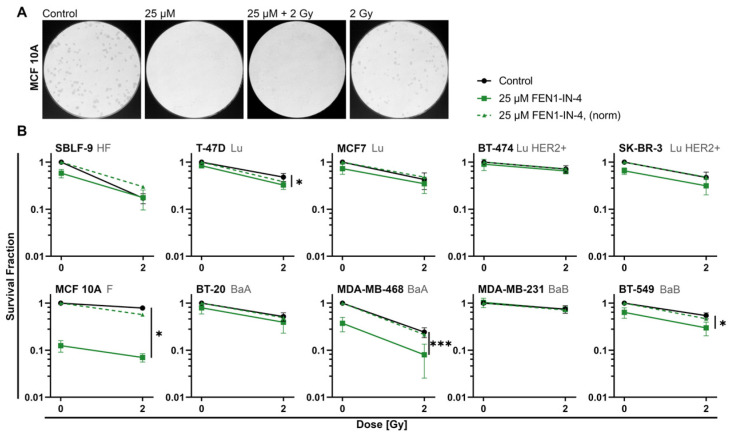
Colony formation assay (CFA) of breast cancer cell lines and non-malignant cell lines with FEN1-IN-4 treatment alone or combined with ionizing radiation (IR). (**A**) Qualitatively assessable images of the Petri dishes of the CFA with MCF 10A. A representative image is shown each for the control, treatment with FEN1-IN-4, IR, or combined therapy, which shows the effects of treatment on colony number and size of this cell line. (**B**) Breast cancer cell lines are grouped according to hormone receptor status, next to mammary epithelial cells (MCF 10A) and healthy skin fibroblasts (SBLF-9) serving as control. The graphs show cell survival as the survival fraction at 0 Gy and 2 Gy either with additional treatment with FEN1-IN-4 or without. To detect synergistic effects between FEN1-IN-4 and IR, the survival fraction was normalized to control, and the normalized cytotoxic effect of the inhibitor alone is shown as a dashed line. Each of the at least four independent experiments contained at least two replicates and their mean value is displayed. Standard deviation is indicated by error bars. Significance between combined treatment and IR was determined by the two-tailed Mann–Whitney U test: * *p* < 0.05, *** *p* ≤ 0.001.

The CFA enabled the determination of the survival fraction, which revealed clear differences between the cell lines (Figure 3B). To detect synergistic, additive, or antagonistic interaction between IR and inhibitor, the values were normalized to the control to indicate the combined effect (Figure 3B, dashed lines). Healthy fibroblasts responded to monotherapy with either IR or inhibitor, whereas with combination therapy cell survival did not decrease further than with IR alone. The normalization indicates an antagonistic effect of FEN1-IN-4 on IR. Some breast cancer cell lines showed little response to both inhibitor and IR (MDA-MB-231, BT-474), others were only mildly affected (MCF7, BT-20). The effect of combined therapy was clearly observable increasing in T-47D (*p* = 0.04), SK-BR-3, BT-549 (*p* = 0.016), and MDA-MB-468 (*p* = 0.001). Except for SK-BR-3, additionally synergistic effects between FEN1-IN-4 and IR were observed in the latter. The strongest influence of the inhibitor alone as well as combined with IR was noted in MCF 10A (*p* = 0.029) with additional synergistic effect to IR. Colony sizes of MCF 10A were also remarkably reduced after monotherapy with FEN1-IN-4 and combination therapy (Figure 3A). The other cell lines did not show this effect and are therefore not shown.

### 2.3. FEN1-IN-4 Induces Cell Death by Apoptosis and Necrosis

**Figure 4:**
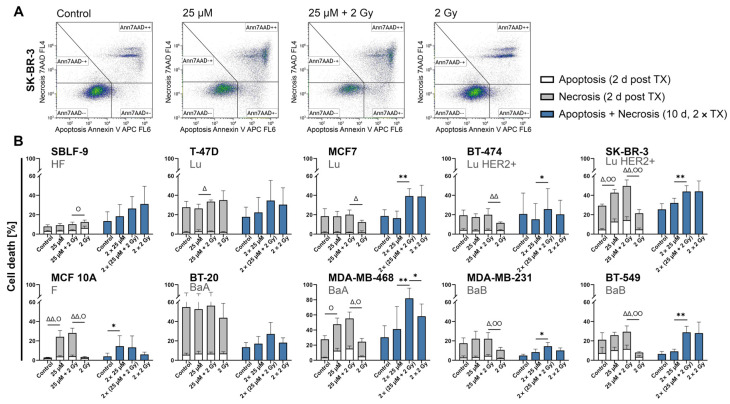
Cell death analysis of malignant and non-malignant cell lines. Cells were treated with either FEN1-IN-4, IR, or both. The response to treatment can be detected by the individual cell death rate in the form of necrosis and apoptosis. In the 10-day protocol, no differentiation is made between apoptosis and necrosis, but the sum is shown. (**A**) Exemplary presentation of the gating strategy of the APC Annexin V and 7-AAD staining applied to SK-BR-3. (**B**) Breast cancer cell lines are grouped by hormone receptor status, next to mammary epithelial cells (MCF 10A) and healthy skin fibroblasts (SBLF-9). TX, treatment. The columns on the left side of each graph display data measured 48 h after a single treatment, while the columns on the right side show the measurement data after a double treatment (day 1, day 5) on day 10. In the measurement on day 10, the sum of apoptosis and necrosis is shown as cell death. Significance calculation was performed with the two-tailed Mann–Whitney U test. Standard deviation is shown by error bars. At least four independent experiments are displayed as the mean values. Δ symbolizes significance of necrosis, O represents significance of apoptosis, and * illustrates significance of combined cell death: Δ/O/* *p* < 0.05, ΔΔ/OO/** *p* < 0.01.

Apoptosis and necrosis were measured with APC Annexin V and 7-AAD staining to investigate influence on cell survival in more detail (Figure 4). “Cell death” means the sum of apoptosis and necrosis, unless otherwise described and combination therapy means combined treatment with FEN1-IN-4 and IR. In the 10-day protocol, no distinction was made between apoptosis and necrosis, but their sum is shown as cell death, as we found apoptosis only makes a small contribution due to the long incubation period. A comparison was made between measurement data of the single treatment in the 2-day protocol and those of the double therapy (day 1 and day 5) in the 10-day protocol (Figure 4B). Overall, a cell line-specific effect was observed with a tendency for the combined therapy to effectively increase cell death in most of the cell lines both in single and double treatment. It is noteworthy that the healthy fibroblasts (SBLF-9) were hardly affected by single treatment whereas double therapy increased cell death in all conditions. The non-malignant mammary epithelial cells (MCF 10A) responded strongly to inhibitor treatment but not to IR. The FEN1-IN-4 monotherapy in single treatment significantly induced more cell death compared to control by necrosis in MCF 10A (*p* = 0.008), SK-BR-3 (*p* = 0.016) and apoptosis in MCF 10A (*p* = 0.016), SK-BR-3 (*p* = 0.008), MDA-MB-468 (*p* = 0.029). These tendencies remained constant after double therapy, for instance in MCF 10A (*p* = 0.015). Compared to IR in single treatment, combined treatment significantly increased both necrosis SK-BR-3 (*p* = 0.008), BT-549 (*p* = 0.008), MCF 10A (*p* = 0.008), MDA-MB-231 (*p* = 0.016), MDA-MB-468 (*p* = 0.029) and apoptosis SK-BR-3 (*p* = 0.008), BT-549 (*p* = 0.008), MCF 10A (*p* = 0.032), MDA-MB-231 (*p* = 0.008), MDA-MB-468 (*p* = 0.029). Those trends were also observed after double therapy, for instance in MDA-MB-468 (*p* = 0.011). Overall, both apoptosis and necrosis occurred as types of death, but necrosis was always the much more frequent contributor to total cell death.

### 2.4. FEN1-IN-4 Induces Senescence

**Figure 5:**
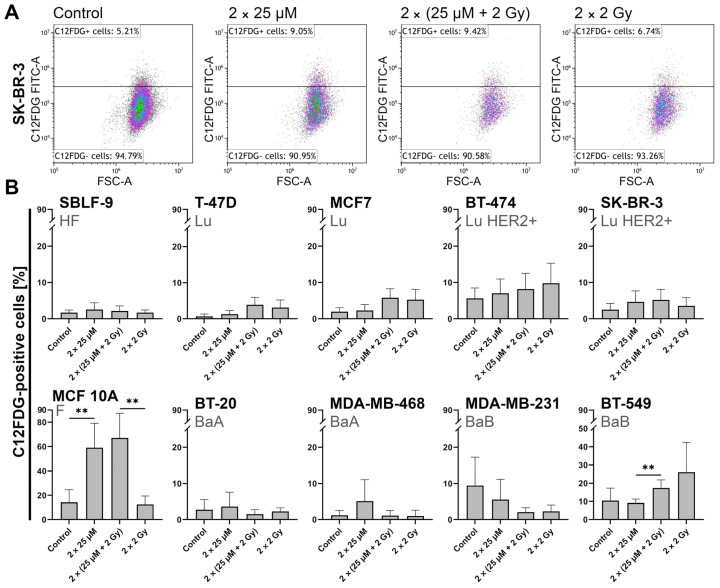
Indication of senescence by relative quantification of C12FDG-positive cells after 10 days incubation period after double therapy with FEN1-IN-4, IR, or both. (**A**) Example of the gating strategy applied to SK-BR-3. Each treatment condition is shown in a separate gate. Each cell line had a specific gate that was adapted to the control and thus transferred to all conditions and experiments. Previously, the cell death gating strategy (Figure 4A) and cell cycle gating strategy (Figure 6A) had been used, so the gates here refer only to living cells in G0/G1-, S-, G2/M phase. (**B**) Sorted according to the hormone receptor status, breast cancer cells are shown next to healthy skin fibroblasts and non-malignant epithelial breast cells. The mean value of at least five independent experiments is displayed. Standard deviation is shown by error bars. The significance was calculated with the two-tailed Mann–Whitney U test: ** *p* < 0.01.

Since the effects in the CFA were different from those of apoptosis and necrosis after one-time treatment, we investigated whether the inhibitor induced senescence in the cells (Figure 5). Thus, we examined whether the more severely reduced survival fraction in T-47D, MCF7, BT-474, and SBLF-9 was also impacted by senescence processes. FEN1-IN-4 increased the proportion of C12FDG-positive cells in all cell lines apart from MDA-MB-231 and BT-549. IR also seemed to trigger this in BT-549, BT-474, T-47D, and MCF7. Interestingly, in MCF 10A, FEN1-IN-4 treatment approximately quadrupled the proportion compared to control (*p* = 0.004) and combination therapy quintupled the proportion of IR alone (*p* = 0.002).

### 2.5. FEN1-IN-4 Influences the Cell Cycle and Increases the G2/M Share

**Figure 6:**
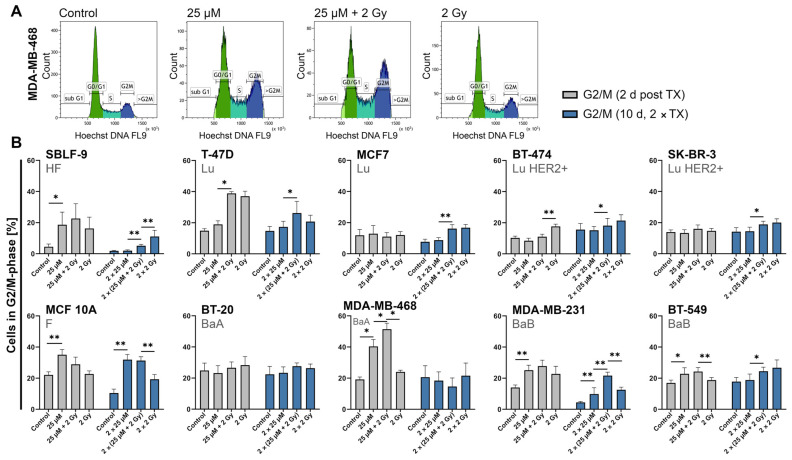
Gating of the cell cycle and percentage of cells in G2/M phase after treatment with FEN1-IN-4, IR, or combined therapy. (**A**) MDA-MB-468 as an exemplary representation of the cell cycle gating strategy, stained with Hoechst 33342. (**B**) Breast cancer cells sorted by hormone receptor status next to healthy fibroblasts and non-malignant breast cells. The left group of columns in diagram shows the data 48 h after single therapy, whereas the right group of columns presents the results after double therapy (day 1, day 5) on day 10. The data correspond to the mean value of at least four independent experiments. Error bars represent the standard deviation. Significance was determined by the two-tailed Mann–Whitney U test * *p* < 0.05, ** *p* < 0.01.

The 2-day protocol with single therapy was compared with the 10-day protocol with double therapy (days 1 and 5) (Figure 6). The complete cell cycle distribution including G0/G1, S, and G2/M is displayed in the supplement material (Supplementary Figures S2 and S3). A decrease in the G0/G1 fraction with a comparable equivalent increase in the G2/M fraction, in the sense of a G2/M arrest, was observed in most cell lines after single treatment. After single treatment with FEN1-IN-4 the G2/M proportion rose approximately 4% in T-47D, 6% in BT-549 (*p* = 0.032), 11% in MDA-MB-231 (*p* = 0.008), 13% in MCF 10A (*p* = 0.008), 14% in SBLF-9 (*p* = 0.029), and 21% in MDA-MB-468 (*p* = 0.029) (Figure 6B). Additional IR strengthened this effect in five cell lines. The G2/M amount remained almost constant regardless of the condition with a single treatment in MCF7, SK-BR-3, and BT-20. BT-474 experienced a significantly higher G2/M fraction by single treatment with IR than with combination therapy (*p* = 0.008), whereas seven other cell lines showed an opposite reaction such as MDA-MB-468 (*p* = 0.029), BT-549 (*p* = 0.008). The proportion of S phase after single treatment was very constant in the respective cell lines, but after treatment with FEN1-IN-4 (with and without IR) in SK-BR-3, it decreased by approximately 30% and in SBLF-9 it significantly tripled (*p* = 0.029 control compared to FEN1-IN-4, *p* = 0.029 2 Gy compared to combined treatment) (Supplementary Figure S2). The cell cycle analysis using the 10-day protocol showed a G2/M arrest in some cell lines that is still present 5 days after the last therapy. The results of both protocols showed similar trends.

### 2.6. FEN1-IN-4 Impedes Population Growth by Extending the Doubling Time

**Figure 7:**
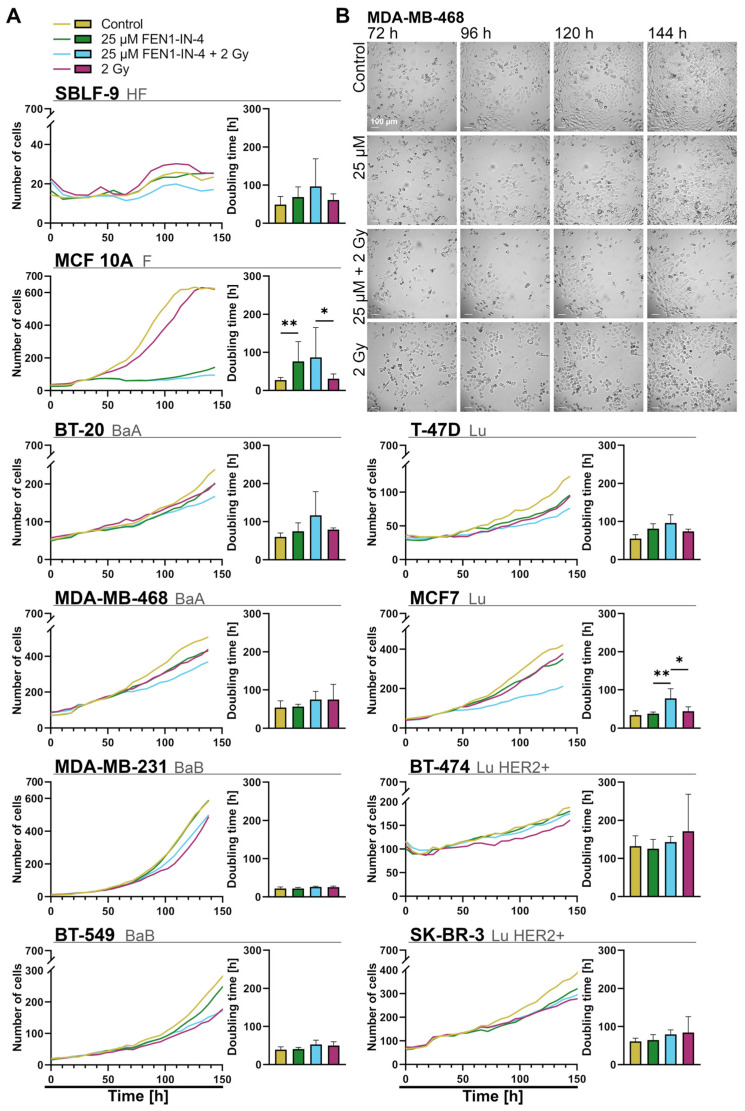
Live-cell microscopy of cells treated with FEN1-IN-4, IR, or both with incubator-microscope system zenCELL Owl. (**A**) Breast cancer cell lines arranged by hormone receptor status, next to healthy fibroblasts and non-malignant breast cells. Automated counting of the cell number enabled the generation of growth curves. The growth curves represent the mean value of at least four individual experiments per cell line with at least two replicates each and their mean values were normalized to the control at the time point of 30 h. By applying an exponential function to each growth curve of the replicates, the doubling time was calculated. The mean of growth curve and calculated doubling time are displayed side by side for each cell line. Error bars indicate standard deviation. Significance was determined by the two-tailed Mann–Whitney U test * *p* < 0.05, ** *p* < 0.01. (**B**) Representative images of MDA-MB-468, showing the influence of FEN1-IN-4, IR, and combination therapy on the growth of cells 72 h, 96 h, 120 h, and 148 h after starting the experiment. The images were captured with zenCELL Owl microscope. The rows show the treatment conditions, and the columns correspond to the recording times. Scale bar represents 100 µm in all images.

Research into population dynamics of cancer cells is an essential basis for deciphering cancer growth [51]. To observe the impact of the therapy regimens on living cells, live cell incubator-microscope system zenCELL Owl was used and the cells were observed for approximately 6 days on an hourly basis. The resulting growth curves (normalized to control after the first 30 h) and doubling times are shown side by side (Figure 7A), accompanied by exemplary images of the MDA-MB-468 cell line (Figure 7B). It was noticeable that the growth curves of the different treatments diverged and fanned out, which was also reflected in the corresponding rising doubling times. The cell lines T-47D, MCF7, MDA-MB-468, and BT-20 showed a tendency for monotherapy with IR or FEN1-IN-4 to have similar retarding effects on growth curves, but this was notably exceeded by combination therapy. In a similar manner, the doubling times of these cell lines rose, comparing combination therapy to IR MCF 10A + 56 h (*p* = 0.032), BT-20 + 37 h, MCF7 + 33 h (*p* = 0.032), and T-47D + 22 h. BT-474 and MDA-MB-231 were decelerated by IR. In BT-549 and SK-BR-3 both FEN1-IN-4 and IR slowed growth in monotherapy only slightly, while combination therapy did not enhance this. The cells of MCF 10A irradiated alone grew almost as much as the control, only slightly delayed. With the addition of FEN1-IN-4, the doubling time of MCF 10A showed a significant raise (monotherapy FEN1-IN-4 compared to control *p* = 0.008; combination therapy compared to IR *p* = 0.032) and hardly any growth occurred. The fibroblasts showed a variegated growth pattern with increased doubling time in all treatments, especially when FEN1-IN-4 was included.

### 2.7. FEN1-IN-4 Leads to DNA Damage

**Figure 8:**
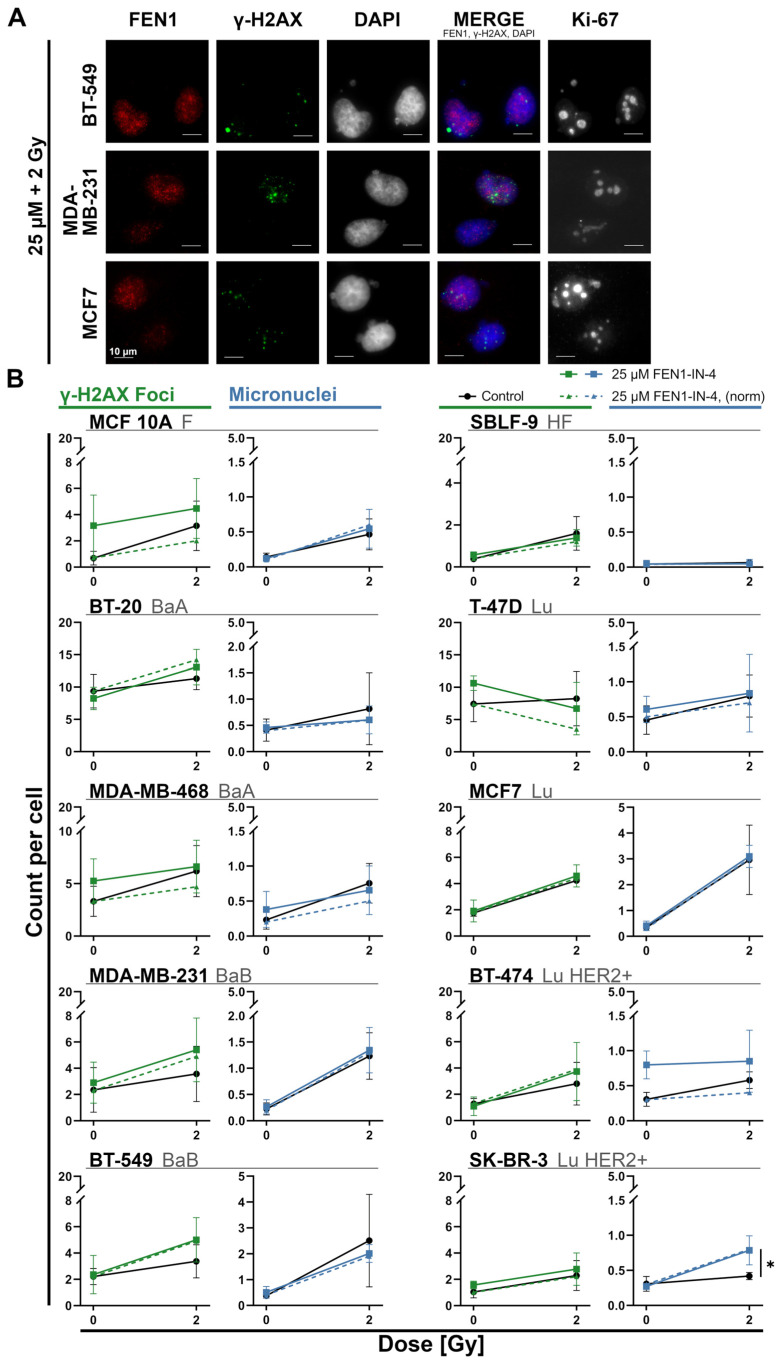
Remaining DNA damage such as γH2AX foci and micronuclei (MN) 24 h after treatment with FEN1-IN-4, IR, or combination of both. (**A**) Exemplary images of immunostaining to visualize remaining DNA damage. Rows show the distinct cell lines treated with combination therapy. FEN1 (red) and γH2AX foci (green) are displayed in the first and second column. DAPI (blue) staining in column three makes cell morphology and MN visible. DAPI, FEN1, and γH2AX foci are merged in column four next to cell proliferation marker Ki-67 in column five. Scale bars represents 10 µm. (**B**) Evaluation of four independent experiments allowed the calculation of the average number of γH2AX foci and MN per cell, which are presented side by side for each cell line. Each cell line has two diagrams displayed directly next to each other, the left one in green represents the γH2AX foci number, the right one in blue represents the MN number. Error bars indicate the standard deviation. Normalization to the control is drawn as a dashed line to show cytotoxicity of FEN1-IN-4. Cell lines are arranged to their hormone receptor status next to healthy skin fibroblasts and non-malignant breast cancer cells. The significance between IR and the combination therapy was computed using the two-tailed Mann–Whitney U test: * *p* < 0.05.

The exemplary images (Figure 8A) show MN and γH2AX foci in three cell lines, including MN with and without γH2AX enrichment. To assess the susceptibility of the DNA as well as repair capacity of the cell lines in general and the DNA damaging effect of the treatment, γH2AX foci and MN were counted by using fluorescence microscopy (Figure 8B). The cytotoxic capacity of FEN1-IN-4 was shown by the normalization of MN and γH2AX foci to the respective control and is displayed as a dashed line. The baseline micronuclei count in the control ranged on average between 0.04 and 0.45 per cell. In all cell lines the MN number increased after IR, in MCF7 the quantity was increased almost tenfold, BT-549 and MDA-MB-231 sixfold, and MCF 10A threefold. However, additional treatment with FEN1-IN-4 had an enhancing effect on the MN number for BT-474, MCF7, T-47D, MDA-MB-231, MCF 10A, and SK-BR-3 (*p* = 0.029). FEN1-IN-4 monotherapy rose both MN (T-47D, MDA-MB-468, and BT-474) and γH2AX foci (T-47D, MDA-MB-468, MDA-MB-231, SK-BR-3, and MCF 10A) number. All cell lines had more γH2AX foci after IR. This could be intensified by combination therapy. Both treatment components induced synergistic increase in MN in BT-20, MDA-MB-231, MCF7, BT-474, and BT-549.

### 2.8. Association between FEN1 Level and Prognosis

**Figure 9:**
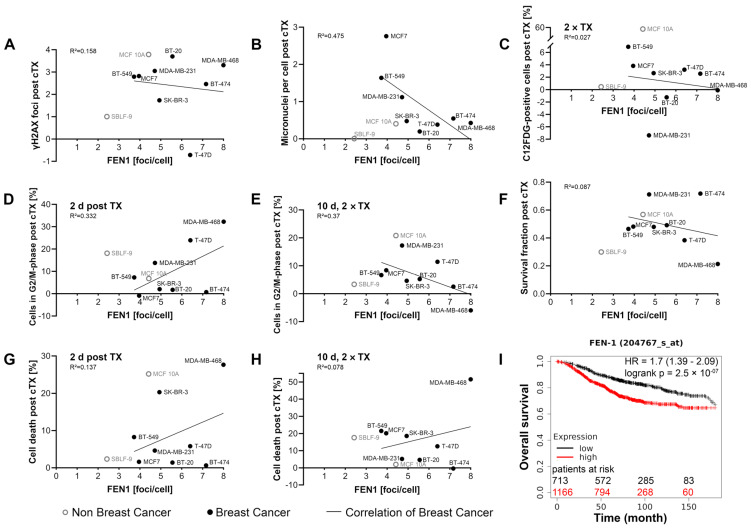
Association of FEN1 level and response to FEN1-IN-4 and IR treatment. (**A**–**H**) The diagrams show a linear regression analysis of the different experimental modalities with combined treatment with FEN1-IN-4 and IR in relation to the FEN1 foci base level of untreated cells. The respective baseline values of the control were subtracted from those of the combination therapy. The analysis was only performed with tumor cells, the non-malignant cell lines are shown in a different color for comparison. Line represents linear regression and the mean value of at least four independent experiments is displayed. cTX, combined treatment. (**I**) Adapted from http://kmplot.com/analysis (accessed on 28 November 2023). A Kaplan–Meier plot comparing the overall survival of patients with breast cancer with either high or low FEN1 mRNA expression. A total of 1879 patients of the Kaplan–Meier plotter database were grouped to a “high” (n = 1166) and a “low” (n = 713) group according to the expression of FEN1 mRNA (Affy ID 204767_s_at) and were examined for 180 months.

Linear regression analyses were performed on the breast cancer cell lines to link the results of the experimental modalities with the previously basic FEN1 quantity. The analysis was carried out with FEN1-IN-4 monotherapy and combination therapy and is shown in Figure 9 and Supplementary Figure S4. DNA damage after combined treatment was negatively correlated to high FEN1 level, as there were slightly less remaining γH2AX foci (Figure 9A) and MN (Figure 9B) were reduced. In contrast, after FEN1-IN-4 monotherapy the γH2AX foci and MN level rose with FEN1 foci number (Supplementary Figure S4A,B). The proportion of C12FDG-positive cells was negatively associated with the foci number after combined treatment (Figure 9C), whereas FEN1-IN-4 monotherapy showed an opposite tendency (Supplementary Figure S4C). The effects on the cell cycle were opposite, the proportion of cells in the G2/M phase after single therapy correlated positively with the foci number (Figure 9E and Supplementary Figure S4E), whereas the same correlated negatively after double therapy and 10 d incubation times (Figure 9D and Supplementary Figure S4F) with either combined treatment or FEN1-IN-4 monotherapy. Cells with high FEN1 foci level had increased cell death after single and double therapy (Figure 9G,H) and reduced the survival fraction (Figure 9F) after combined treatment. The same applied to cell death (Supplementary Figure S4G,H) and the survival fraction after FEN1-IN-4 monotherapy (Supplementary Figure S4D). Apoptosis and necrosis in general were also increasing with FEN1 foci count (Supplementary Figure S4I–L). To transfer the effect of FEN1 quantity to the clinical context, the relationship between FEN1 expression and survival was investigated. A total of 1879 breast cancer patients of the Kaplan–Meier plotter database (http://kmplot.com/analysis) (accessed on 28 November 2023) were divided into the “high” and “low” groups based on the FEN1 mRNA expression and the overall survival was presented in a Kaplan–Meier plot (Figure 9B) [52,53]. The Kaplan–Meier plot showed a significantly poorer overall survival for patients with high FEN1 mRNA expression. The log-rank test showed an increased hazard ratio (HR) of 1.7 (range 1.39–2.09) and indicates a lower survival probability for patients with high FEN1 expression (*p* = 2.5 × 10^−7^) as the upper quartile survival for the low-expression cohort was 143.74 months and for the high-expression cohort 68.4 months.

## 3. Discussion

Breast cancer, especially TNBC, is often chemoresistant and new treatment options are highly required [14]. This study examined the effects of the FEN1 inhibitor FEN1-IN-4 on breast cancer cell lines including four TNBC cell lines and whether it is a useful therapeutic agent in breast cancer therapy in monotherapy or in combination with IR. Overall, the results showed a cell line-specific response to treatment with FEN1-IN-4 or combined therapy with IR. Apparently, there are cells that are more sensitive or resistant to therapy with FEN1-IN-4. This could also be due to mutations in the FEN1 gene, as it is well known that breast cancer genetic subtypes are very heterogenous [5]. FEN1-IN-4 can exert its impact via cytotoxic, cytostatic, and radiosensitizing effects.

### 3.1. FEN1 Distribution and Variance in Cells

FEN1 has a dynamic distribution within the cell depending on the cell cycle, during G1 phase it is translocated in to the nucleus and in G2 back to the cytoplasm and it is hypothesized that it is also recruited to the nucleus by DNA damage [31]. It is also known that a mitochondrial isoform of FEN1 exists [54]. FEN1 is usually distributed in cytoplasm and nucleus of cells, and it is known to superaccumulate in nucleoli [27,31]. This accumulation was also present in T-47D, BT-20, MDA-MB-468, and MCF 10A in this study. The FEN1 quantity was determined approximately by FEN1 foci counting. The cell lines had heterogenous amounts of baseline foci to which the FEN1 intensity in the nucleus and cytoplasm were positively correlated.

### 3.2. FEN1-IN-4 Has Cytotoxic Effects

FEN1-IN-4 monotherapy induced cytotoxicity through apoptosis and necrosis in five out of ten cell lines (three of them TNBC; MCF 10A, SK-BR-3, MDA-MB-468, MDA-MB-231, and BT-549), which was enhanced by IR, and these tendencies of cell death could also be similarly reproduced after double therapy and 10 days of incubation in four of these cell lines (three of them TNBC; SK-BR-3, MDA-MB-468, MDA-MB-231, and BT-549). Three other cell lines developed a similar pattern regarding cell death only after double therapy (BT-20, T-47D, and SBLF-9) whereas another one seemed to be influenced only by IR (MCF7). The development of increased cell death after double therapy and the prolonged incubation time could also indicate long-term effects of the inhibitor, which would require investigation in more detail. Cell death by monotherapy and combination therapy via necrosis and apoptosis was associated to the initial amount of FEN1. However, the cell lines that suffered only minor additional cell death as a result of monotherapy or combination therapy were not completely unaffected, as the treatment could apparently also exert its effect by other mechanisms. This is manifested by various response patterns in the form of remaining DNA damage, prolonged doubling times, shifts in the cell cycle to G2/M arrest, cellular senescence, and reduced the survival fraction.

The cytotoxicity of FEN1-IN-4 is also indicated by a reduced survival fraction and FEN1-IN-4 can also support the effect of IR. Our results regarding the survival fraction were similar in trend to another study with the FEN1 inhibitor C8, which reported an increasing survival fraction after inhibitor therapy in the following cell lines: MDA-MB-468 < BT-549 < T-47D < SK-BR-3 < MCF7 < MDA-MB-231 [55].

In radiotherapy, the cell-killing effect of IR is based on DNA damage, such as DSB and cell death correlates very closely with DSB breaks [28,56]. Remaining DSBs after radiation exposure are assumed to be the main cause of reduced cell survival [57]. As DSB are the most severe forms of DNA damage, cells immediately repair those lesions to prevent cell death and carcinogenesis [29,58]. To do this, proliferating cells also stop the cell cycle [58]. γH2AX foci visualize remaining DSB after treatment [59]. It is unfavorable for the efficacy of radiotherapy that the previously underlying FEN1 foci number was negatively associated with number of γH2AX foci after combined treatment and thus the amount of DSB in the cell lines with high FEN1 levels was lower than in others with initially less FEN1. It is encouraging that the combination with FEN1-IN-4 was able to synergistically increase the γH2AX foci count by IR in eight out of ten cell lines. Therefore, FEN1-IN-4 might enhance the DNA-damaging effect of IR, and this could indicate radiosensitization.

MN can be induced by DNA breaks caused by drugs or radiation and are therefore also considered a “hallmark of genotoxic stress” [60]. MN are already classified as biomarker and indicator of genotoxicity and chromosome instability [61,62,63]. MN frequency, as a sign of sensitivity to mutagens, has already been considered as a factor in the assessment of individual breast cancer risk [64]. Since cancer therapies are based on the generation of damage in cells, the activity and specificity of the treatment could be revealed through the increased number of MN [65]. A highly variable baseline amount of MN in untreated cell lines was observed, but in almost all of them an increase could only be provoked by IR. However, in one cell line (SK-BR-3), MN numbers rose significantly through combined therapy compared to IR.

### 3.3. FEN1-IN-4 Affects the Cell Cycle

Cytotoxicity might also be expressed by G0/G1 and G2/M arrest [66]. The fact that apoptosis follows G2/M arrest has already been discovered in lung cancer cells [67]. Other studies have already presented new potential drugs against breast cancer that develop their effect through G2/M arrest and apoptosis [68]. To have the chance to repair DNA after genotoxic damage, DNA checkpoints are activated, such as the G2/M checkpoint before mitosis [69]. Therefore, DNA damage can lead to temporary cell cycle inhibition to enable repair [33]. This might be the link between cytotoxicity and cell cycle arrest in FEN1-IN-4 treatment. Usually, cancer cells with a defective G1 checkpoint nevertheless have an intact function of the G2 checkpoint [70]. We expected an intact cell cycle regulation in healthy fibroblasts, which was also supported by an increased S fraction after FEN1-IN-4 monotherapy and combination therapy. FEN1-IN-4 led to an increase in G2/M proportion in six out of ten cell lines (three of them TNBC; T-47D, MDA-MB-468, MDA-MB-231, BT-549, MCF 10A, and SBLF-9) and was further potentiated by IR in five of the six (three of them TNBC; T-47D, MDA-MB-468, MDA-MB-231, BT-549, and SBLF-9). This could indicate a G2/M arrest. This could be an advantage for combined radiotherapy, as the G2 phase is a more radiosensitive cell cycle phase [71]. Prolonged G2/M arrest was also observed after double therapy and 10 days incubation period. Furthermore, other studies have shown that FEN1 inhibitor sensitive cell lines enter G1 and or G2/M arrest due to reduced DNA replication capacity [55].

Due to the discrepancy between the reduced survival fraction and induced cell death in some cell lines (T-47D, MCF7), it was determined whether FEN1-IN-4 initiates cellular senescence. Cellular senescence can be described as the definite exit from the cell cycle [72]. These cell lines actually showed an increased proportion of C12FDG-positive cells. Apoptosis and cellular senescence are relevant pathways to restrain pathologic proliferation of cells [68]. However, the senescence-promoting effect of FEN1-IN-4 also only appears to influence certain cell lines. The striking significant increase in MCF 10A remains unclear.

### 3.4. FEN1-IN-4 Has a Cytostatic Influence on Cells

The prolongation of the doubling times shows the cytostatic effects of FEN1-IN-4 and the restricted growth of the population after treatment with FEN1-IN-4. Although in the intercellular comparison some cell lines only experienced a low cell death rate, they were strongly inhibited in growth by a prolonged doubling time by + 56 h for BT-20, by + 43 h for MCF7, and by + 41 h for T-47D after combined treatment. This could also constitute a further therapeutic strategy in the sense of tumor progression control.

### 3.5. The Impact of FEN1-IN-4 on Non-Malignant Cells

Interestingly, the non-malignant epithelial cells of a fibrocystic breast (MCF 10A) were particularly susceptible to therapy with FEN1-IN-4, whereas IR alone or in addition had only a minor further effect. The healthy skin fibroblasts (SBLF-9) were examined to simulate the reaction of normal tissue to FEN1-IN-4 and IR. The fibroblasts only had a moderate response to FEN1-IN-4 monotherapy or combined with IR. The treatments hardly triggered any cell death, and in the CFA, the combination therapy was not able to reduce the survival fraction any further than IR alone. However, the doubling time was extended, and the G2/M fraction was also increased. This might be an indication of possible side effects during course of the therapy.

### 3.6. Is FEN1 a New Potential Biomarker for the Treatment of Breast Cancer?

Breast cancer markers support early diagnosis, assess the effectiveness of treatment, and then select treatment concepts [73]. However, the biomarkers and therapeutic targets used to date neglect other breast cancer mutations that have already been discovered [1]. Unfortunately, there is also a shortage of appropriate biomarkers in research to choose patients for treatment strategies according to their oncogenic drivers [74]. FEN1 could serve as a tumor marker to detect patients with a high probability of progression and to predict the outcome [25]. Although the FEN1 gene is considered a tumor suppressor gene, increased expression cannot prevent carcinogenesis [75]. It is already known that DNA methylation correlates with prognosis in breast cancer patients [75]. FEN1 levels are strictly regulated in human cells, usually FEN1 contents are low in healthy breast cells due to hypermethylation of the FEN1 promoter [45]. Hypomethylation of FEN1 promotor is already linked to overexpression of FEN1 [25]. Studies showed that FEN1 expression is increased 2.5-fold in breast cancer tissue and the FEN1 expression is even higher in metastatic tissue than in the associated tumor [25]. This study of FEN1 expression showed a similar trend with a 1.3-fold median FEN1 gene expression in breast cancer tumor and a 1.7-fold median FEN1 gene expression in metastatic tissue compared to normal breast tissue [50]. In addition, the healthy fibroblasts had lower levels of FEN1 foci than the breast cancer cells, which had much higher FEN1 foci count and FEN1 intensity levels.

### 3.7. Is FEN1 Relevant for Prognosis?

To continually improve therapy, it is necessary to find the genes that influence cancer progression and outcome [25]. In fact, the Kaplan–Meier plotter database showed a significant tendency for patients with highly expressed FEN1 mRNA to have a worse overall survival than with lower expression [52,53]. Consequently, and as already suspected, FEN1 may play an important role as a prognostic marker in the future [47]. It has already been hypothesized that the level of expression can be directly correlated with grading and staging of breast cancer [25]. The enhanced expression of FEN1 could be linked to the accelerated proliferation rate in cancer cells and be required for BER in the case of DNA damage [25]. Enhanced FEN1 expression in cancer cells improves DNA repair processes and can lead to the development of drug resistance [45]. Indeed, the cell lines that had a high FEN1 level before treatment had less DNA damage in the form of remaining DSB and MN after combination therapy. This could be the result of the enhanced repair capacity and provoke treatment resistance.

### 3.8. Is FEN1-IN-4 a New Potential Targeted Therapeutic Agent for Breast Cancer?

However, patients could benefit from targeted therapy, which is why further development in these fields is needed [1]. Since tumors are heterogenous, it is necessary to provide different chemotherapeutic regimens [76]. FEN1 might be a therapeutic target due to its function at the cellular level, its elevated gene expression in breast cancer, and its association with drug resistance [45]. FEN1 has already been described as a potential target structure for inhibitor-based therapy of cancer cells, particularly those with defects in homologous recombination [55]. Several different approaches to FEN1 inhibition have already been established, including small interfering RNAs (siRNA) to reduce the FEN1 protein level and N-hydroxy urea FEN1 inhibitors [55,77]. But FEN1 inhibition also faces limiting challenges. It is already known that distinct cell lines have heterogenous levels of sensitivity to various FEN1 inhibitors: BRCA1 and BRCA2 mutants, for example, are generally more sensitive, while MDA-MB-231 is resistant to the FEN1 inhibitor C8 [55]. Other studies report cell lines that were able to regenerate after the end of FEN1 inhibition and were stable against death from the treatment [55]. Nonetheless, even incomplete FEN1 inhibition and the resulting possibly impaired interaction and complex formation with other proteins could also lead to DNA damage, disruption of replication, and cell cycle arrest [55]. FEN1-IN-4 induced the highest cell death rate in the cell line with the highest foci number (MDA-MB-468). The cell line with the second highest number of foci (BT-474) suffered only little cell death. Consequently, concerns about FEN1 level-dependent effects may be refuted, or there may be other additional factors influencing this association. And this could again be an indication of a cell-specific response to therapy, which is nevertheless an option regarding personalized medicine. FEN1 inhibition is attributed to sensitization to DNA damage that is usually repaired by BER [77]. FEN1-IN-4 could also help control tumor progression in cancer therapy by slowing population growth, generating DSB and triggering senescence and cell death. In addition, by inducing a G2/M arrest, FEN1-IN-4 could be a useful adjunct to radiotherapy to bring cancer cells into a radiosensitive phase, thereby improving the efficacy of radiotherapy [71]. Since this study revealed that, notably, three out of four TNBC and other breast cancer cell lines show a good response to FEN1-IN-4 monotherapy and combination therapy with IR, FEN1-IN-4 may expand the therapeutic spectrum as a potential target therapy. An important question that needs to be investigated through further studies is how either increased resistance or sensitivity to FEN1 inhibition occurs.

## 4. Materials and Methods

### 4.1. Cell Lines and Cell Culture

Ten cell lines were used, of which eight were breast cancer cell lines (T-47D, MCF7, BT-474, SK-BR-3, BT-20, MDA-MB-468, MDA-MB-231, and BT-549) next to non-malignant mammary epithelial cells (MCF 10A) and healthy skin fibroblasts (SBLF-9). MCF7, BT-20, BT-549, and MDA-MB-231 were purchased from CLS Cell Lines Service (Eppelheim, Germany). T-47D, BT-474, SK-BR-3, MDA-MB-468, and MCF 10A were kindly provided by Matthias Rübner (Gynecological Clinic of University Hospital Erlangen, Erlangen, Germany). The primary fibroblast cell line SBLF-9 was established from cells of a healthy donor in our laboratory. The cells were cultured in a specific nutrient medium with additional 10% fetal bovine serum (FBS, Sigma-Aldrich, St. Louis, MI, USA) and 1% penicillin-streptomycin (Thermo Fisher Scientific, Waltham, MA, USA) unless stated otherwise. They were passaged every 2–3 days to a maximum of 50 passages in a humidified atmosphere at 37 °C and 5% CO_2_. T-47D was cultivated in RPMI-1640 Medium (Sigma-Aldrich, St. Louis, MI, USA) supplemented with 10 µg/mL recombinant human insulin AOF (Thermo Fisher Scientific, Waltham, MA, USA). MCF7 was cultured in Dulbecco’s Modified Eagle’s Medium (DMEM; PAN-Biotech GmbH, Aidenbach, Germany). BT-474 received DMEM complemented with 0.02 mM L-Glutamine (Thermo Fisher Scientific, Waltham, MA, USA), 0.005 g/L Glucose Solution (Thermo Fisher Scientific, Waltham, MA, USA), and 17.8 mM sodium bicarbonate solution (NaHCO_3_; Sigma-Aldrich, St. Louis, MI, USA). SK-BR-3 was cultivated in McCoy’s 5A Medium (1X) Modified with L-Glutamine (Thermo Fisher Scientific, Waltham, MA, USA). For cultivation of BT-20 and MDA-MB-468 Dulbecco’s Modified Eagle Medium/Nutrient Mixture F-12 (Ham) 1:1 + L-Glutamine + 2.438 g/L Sodium Bicarbonate (Thermo Fisher Scientific, Waltham, MA, USA) was used. MDA-MB-231 was cultured in DMEM, BT-549 received Dulbecco’s Modified Eagle Medium (1X) + GlutaMAX^TM^-I with 4.5 g/L D-Glucose (Thermo Fisher Scientific, Waltham, MA, USA). The medium for MCF 10A consisted of Mammary Epithelial Cell Growth Medium and associated SupplementMix (both from PromoCell GmbH, Heidelberg, Germany) and 100 ng/mL Cholera Toxin from *Vibrio cholerae* (Sigma-Aldrich, St. Louis, MI, USA) without FBS. For SBLF-9 culture F-12 Nutrient Mixture (HAM) (1X) (Thermo Fisher Scientific, Waltham, MA, USA), 15% FBS, and 2% non-essential amino acids (Bio&SELL GmbH, Feucht, Germany) was used.

To enable clinical and scientific visualization of the results, the cell lines were classified according to the following nomenclature (Table 1). All breast cancer cell lines are BRCA1 wild type [78].

### 4.2. Treatment with Inhibitor FEN1-IN-4 and Ionizing Radiation

FEN1 inhibitor FEN1-IN-4 (Selleck Chemicals LLC, Huston, TX, USA) was dissolved in dimethyl sulfoxide (DMSO; Carl Roth GmbH + Co. KG, Karlsruhe, Germany) and stored in aliquots at −80 °C. During experiments, the required amount was thawed immediately before use. A concentration of 25 μM was selected for the experimental setup.

The irradiation of cells with 2 Gy of IR was performed by an ISOVOLT Titan X-ray generator (GE, Ahrensburg, Germany). In all experiments, there were four experimental conditions. Cells were treated either with 2 Gy of IR or 25 μM FEN1-IN-4 or a combination therapy of both. For the combination therapy the cells were irradiated 3 h after treatment with the inhibitor. The control group was treated analogously only with DMSO, unless otherwise noted.

### 4.3. Imaging DNA Damage, Micronuclei, Cell Proliferation, and FEN1 by Immunofluorescence Microscopy

Cells were simultaneously seeded in separate 8-well silicone chambers (flexiPERM, Sarstedt, Nümbrecht, Germany) on glass slides and cultured for 48 h to allow growth. After a change in medium, they were treated with the different conditions and incubated for another 24 h. In contrast to the other experiments, here the control was not treated with DMSO. After the cells were fixed and permeabilized for 15 min with 4% formaldehyde solution (Sigma-Aldrich, St. Louis, MI, USA), the slides were blocked with 1% bovine serum albumin (BSA; SERVA Electrophoresis GmbH, Heidelberg, Germany) and 10% FBS (Sigma-Aldrich, St. Louis, MI, USA) solution overnight at 4 °C in the dark. All antibodies were diluted with a 1% BSA and 1X Tris-Buffered Saline solution. The following primary antibodies were used for staining overnight at 4 °C: mouse anti-FEN1 (1:50; Santa Cruz Biotechnology, Dallas, TX, USA), rabbit anti-γH2AX (1:400; Cell Signaling Technology, Danvers, MA, USA), rat anti-Ki67 (1:100; Invitrogen, Thermo Fisher Scientific, Waltham, MA, USA). The next day, the staining process was continued with the secondary antibodies with an incubation time of 90 min at 20 °C in the dark: Alexa Fluor 555 donkey anti-mouse (1:200; Invitrogen, Thermo Fisher Scientific, Waltham, MA, USA), Alexa Fluor 488 chicken anti-rabbit (1:200; Invitrogen, Thermo Fisher Scientific, Waltham, MA, USA), Alexa Fluor 647 goat anti-rat (1:200; Invitrogen, Thermo Fisher Scientific, Waltham, MA, USA), Alexa Fluor 647 donkey anti-rat (1:200; Abcam, Cambridge, UK). Finally, the DNA was visualized with 4′,6-Diamidine-2′-phenylindole dihydrochloride (DAPI; Sigma-Aldrich, St. Louis, MI, USA) and cells were embedded with Vectashield (Vector Laboratories, Newark, CA, USA) and cover slides were added. Images were captured by a Zeiss Axio Plan 2 fluorescence microscope (Zeiss, Oberkochen, Germany) and software Metafer 4 (MetaSystems, Altlussheim, Germany). Biomas Software (MSAB, Erlangen, Germany) enabled automated image analysis with foci quantification. For visualization of FEN1, image acquisition and analysis were performed with the same parameters to enable a comparison of the distinct cell lines. Micronuclei (MN) were scored from DAPI stained cells according to the following rules: (1) MN are round extra-nuclear structures, (2) MN are positive for DAPI, (3) MN have a similar morphology compared to nuclei, (4) MN are smaller than nuclei, (5) MN can touch nuclei but without a connection, and (6) MN were in close proximity to the cells [76,79,80]. Only cells with normal morphology were analyzed, excluding those in the mitotic phase and apoptotic and necrotic appearing cells [81]. The average number of MN per cell was calculated. By using FEN1 antibody for immunostaining, the quantitative and local distribution of FEN1 is revealed.

### 4.4. Clonogenic Cell Survival by Colony Formation Assay

The gold standard to measure cell reproductive death caused by IR is the colony formation assay (CFA) [82]. The CFA was used to assess the ability of single cells to survive and form colonies despite therapy [83]. At the beginning, a certain number of cells was seeded in small Petri dishes and treated with the four conditions after 18 h. After another 48 h the medium was replaced by fresh medium and the cells were further incubated up to 14 days, depending on the cell line. Once colonies of minimum 50 cells were formed, fixation and staining were performed with methylene blue (Carl Roth GmbH + Co. KG, Karlsruhe, Germany) for 30 min at 20 °C. Subsequently, the colonies were counted using the software Biomas (MSAB, Erlangen, Germany). The plating efficiency was determined in the control based on the proportion of grown colonies of seeded cells. To determine the cell survival after the specific treatment, the respective survival fraction was calculated as the ratio of colonies grown after treatment to the number of cells seeded and was normalized to the plating efficiency. Most experiments were performed with more than two technical replicates per condition.

### 4.5. Combined Assay for Apoptosis, Necrosis, and the Cell Cycle by Flow Cytometry (2-Day Protocol)

On day 0, cells were seeded in T25 flasks and cultured for 24 h. After replacing the medium with 2% FBS culture medium (MCF 10A received again medium without FBS), they were treated with the four conditions and left to grow for 48 h. Cells were analyzed by flow cytometry 48 h after treatment. All cells and the supernatant were harvested and centrifugated (5 min, 20 °C, 300× *g*). The supernatant was discarded, and the cell suspension was divided into two parts. One part for the measurement of apoptosis and necrosis and the other for cell cycle analysis. To determine apoptosis and necrosis, the samples were directly resuspended with 200 μL cold Ringer’s solution (Fresenius Kabi AG, Bad Homburg, Germany) and 10 μL of a 1:1 mixture of Allophycocyanin Annexin V (APC Annexin V, BD Biosciences, Franklin Lakes, NJ, USA) and 7-amino-actinomycin D (7-AAD, BD Biosciences, Franklin Lakes, NJ, USA) were added and samples were incubated for 30 min at 4 °C in the dark.

For cell cycle analysis, cells were resuspended in 1 mL of 2% FBS medium (MCF 10A in 0% FBS) and 10 mL of cold 70% ethanol (Otto Fischar GmbH & Co. KG, Saarbrücken, Germany) and then stored at 4 °C in the dark for at least 24 h. After centrifugation (5 min, 20 °C, 300× *g*) and discarding the supernatant, the sample was diluted with 1 mL of cold Ringer’s solution (Fresenius Kabi AG, Bad Homburg, Germany) and incubated with 3 μL of Hoechst 33,342 (Thermo Fisher Scientific, Waltham, MA, USA) for 1 h at 4 °C in the dark.

Centrifugation and resuspension in Ringer’s solution were performed again before both partial analyses. The CytoFLEX S flow cytometer (Beckmann Coulter, Brea, CA, USA) performed the measurement by using the PB450 channel for the cell cycle, PerCP channel for necrosis, and APC channel for apoptosis. The results were evaluated using Kaluza Analysis software (Beckmann Coulter, Brea, CA, USA). Forward and side scattering identified cells, dividing them in size and granularity. Among the single cells, the necrotic cells were defined as those with positive signal for APC Annexin V and 7-AAD, and apoptotic cells with positive APC Annexin V signal and negative 7-AAD signal. Living cells were defined as APC Annexin V negative and 7-AAD negative. For cell cycle analysis, cells were sorted by DNA content.

### 4.6. Combined Assay for Senescence, Apoptosis, Necrosis, and the Cell Cycle by Flow Cytometry (10-Day Protocol)

Senescent cells enrich ß-galactosidase in lysosomes [84]. After neutralization of the pH in the lysosomes by Bafilomycin A1, ß-galactosidase hydrolyses 5-Dodecanoylaminofluorescein Di-β-D-Galactopyranoside (C12FDG) [84]. Hydrolyzed C12FDG remains in the cell and can fluoresce green when excited and cells can be identified by C12FDG fluorescence signal [84,85]. In conclusion, C12FDG fluorescence and therefore C12FDG positivity can be considered as an indication for senescence [84,86,87]. To give the cells time to become senescent, a treatment regimen was selected with seeding on day 0, treatment on day 1 and again on day 5, and finally measurement on day 10. Before each treatment the medium was exchanged (SBLF-9 received medium with 15% FBS and MCF 10A 0% FBS, other cell lines 10% FBS). Cell analysis was carried out on day 10. After harvesting supernatant and all cells, centrifugation (8 min, 20 °C, 180× *g*), discarding the supernatant, and resuspension in medium (2% FBS, 0% FBS for MCF 10A), the first step was to treat with 4 μL Bafilomycin A1, *Streptomyces griseus* (Sigma-Aldrich, St. Louis, MI, USA) per sample for 30 min. Then, 2 μL Hoechst 33,342 (Thermo Fisher Scientific, Waltham, MA, USA) was added and incubated for 30 min, followed by 0.5 μL C12FDG (Thermo Fisher Scientific, Waltham, MA, USA), and the incubation resumed for another hour (all incubation times so far were carried out at 37 °C and 5% CO_2_ in the dark). Cells were then centrifuged (6 min, 20 °C, 400× *g*), the supernatant discarded, resuspended in 200 μL of Ringer’s solution (Fresenius Kabi AG, Bad Homburg, Germany), and then stained for 30 min with 10 μL of the 1:1 mixture of APC Annexin V (BD Biosciences, Franklin Lakes, NJ, USA) and 7-AAD (BD Biosciences, Franklin Lakes, NJ, USA) shielded from light at 4 °C. Finally, before measurement, the cells were centrifuged again (6 min, 20 °C, 400× *g*), the supernatant discarded, and the pellet resuspended in 150 L Ringer’s solution. Afterwards the measurement was performed by CytoFLEX S flow cytometer (Beckmann Coulter, Brea, CA, USA) using PB450, FITC, PerCP, and APC channels. Kaluza Analysis software was used for evaluation of the results. The gating strategy for the cell cycle, apoptosis and necrosis was applied analogously to the other experiment. Gating of C12FDG staining was restricted to living cells in G0/G1-, S-, G2/M phase. Based on the control, a gate was selected in the FSC and C12FDG fluorescence signal (FITC channel), which was then used for the respective cell line to detect shifts of the populations after different treatments.

### 4.7. Monitoring of Living Cells with 24-Channel Microscopy

During this experiment, the 24-well plate containing the cells was placed in a 24-channel microscope (zenCELL owl, innoME GmbH, Espelkamp, Germany) in the incubator and images were taken every hour for approximately 6 days. For this purpose, the cells were seeded into the plate at low density and image acquisition was started after 1–3 h to allow adherence to the plate, which was necessary for image quality. After 24 h of growth, the cells were treated, but only half of the plate was irradiated. Another 48 h later, the medium was exchanged. Each cell line was studied in 4 individual experiments with at least 2 replicates for each condition. The associated zenCELL software version v3.4.1 (innoME GmbH, Espelkamp, Germany) simultaneously analyzed the captured images and determined the number of cells so that, using an exponential function, the doubling time could be extracted from the growth curve, in the exponential growth zone.

(1) $$y=y0\times \exp\left(k\times t\right)$$

In the exponential function shown above, y0 is the cell number at the start of microcopy, k is the rate constant, and t is time in h. The doubling time is calculated in the following way:

(2) $$doubling\;time=\frac{ln(2)}{k}$$

### 4.8. Statistical Analysis

All experiments were performed at least four times independently. Graphs were drawn with GraphPad Prism 9 (GraphPad Software, San Diego, CA, USA). The data were analyzed by the two-tailed Mann–Whitney U test using GraphPad Prism 9 (GraphPad Software, San Diego, CA, USA). Resulting *p*-values ≤ 0.05 were accepted as significant. To measure the inhibitor’s own strength, we tested the control against monotherapy with FEN1-IN-4. Based on the clinical background, we compared the IR treatment alone with the combination therapy to identify radiosensitizing effects. The combination therapy is also compared with FEN1-IN-4 monotherapy.

### 4.9. Investigation of Prognosis Depending on FEN1 Gene Expression Using the Kaplan–Meier Plotter Database

The overall survival analysis related to FEN1 mRNA (Affy ID 204767_s_at) expression was carried out using the Kaplan–Meier plotter database in its default settings with “auto select best cutoff option”, Kaplan–Meier survival curves were drawn, and the log-rank test was carried out as well as hazard ratio and significance were calculated (http://kmplot.com/analysis) (accessed on 28 November 2023) [52,53]. A total of 1879 patients with breast cancer were included without restrictions regarding specific pathological or molecular characteristics of breast cancer with 180 months follow-up.

### 4.10. Expression of the FEN1 Gene in Breast Tissue, Tumor, and Metastasis Using TNMplot Database

The TNMplot database was used to retrieve data to compare the expression of the FEN1 gene in breast cancer tumors (n = 7569) and normal (n = 1712) and metastatic (n = 82) tissue (https://tnmplot.com) (accessed on 2 December 2023) [50]. Significance was calculated using the Kruskal–Wallis and Dunn’s tests (n = 7893) and violin diagrams were drawn as well by the TNMplot database.

## 5. Conclusions

Overall, FEN1-IN-4 appears to significantly exert its effects through multiple pathways and can significantly induce DNA damage, apoptosis, necrosis, senescence, and G2/M arrest, reduce clonogenic survival and slow cell growth. The effect of FEN1-IN-4 on breast cancer cells, including TNBC, can be achieved both with monotherapy and in combination with IR. There seems to be a tendency for some cell lines to be more sensitive to FEN1 inhibition. FEN1 might represent both a possible diagnostic tool in form of a biomarker and a potential target molecule for a novel personalized therapy in breast cancer.

## Figures and Tables

**Table 1:** Clinical, pathological, and genetic classification of breast cell lines.

Cell Line	Origin	ER	PR	HER2	Gene Cluster
T-47D	IDC	+	+		Lu
MCF7	IDC	+	+		Lu
BT-474	IDC	+	+	+	Lu HER2+
SK-BR-3	AC	-	-	+	Lu HER2+
BT-20	IDC	-	-		BaA
MDA-MB-468	AC	-	-		BaA
MDA-MB-231	AC	-	-		BaB
BT-549	IDC, pap	-	-		BaB
MCF 10A	Fib	-	-		BaB

Classification of breast cell lines based on clinical, pathological, and genetic criteria. Adapted from [5]. AC, adenocarcinoma; BaA, Basal A; BaB, Basal B; ER, estrogen receptor; Fib, fibrocystic disease; HER2, human epidermal growth factor receptor 2; HER2+, HER2 amplified; IDC, invasive ductal carcinoma; Lu, Luminal; Lu HER2+, Luminal HER2 amplified; pap, papillary; PR, progesterone receptor. ER, PR, and HER2 status: ER and PR positivity and HER2 overexpression are indicated as +. - stands for no positivity or overexpression. Empty fields result due to missing information. The original data originate from either protein and or mRNA levels.

## Data Availability

The data generated and presented in this study are obtainable on request from the corresponding author. Publicly available data presented in this study can be accessed on the following websites: KMplot (http://kmplot.com/analysis, accessed on 28 November 2023), TNMplot (https://tnmplot.com, accessed on 2 December 2023).

## Supplementary Materials

The following supporting information can be downloaded at: https://www.mdpi.com/article/10.3390/ijms25042110/s1.

## References

[1] Harbeck N., Gnant M. (2017). Breast cancer. Lancet.

[2] Arnold M., Morgan E., Rumgay H., Mafra A., Singh D., Laversanne M., Vignat J., Gralow J.R., Cardoso F., Siesling S. (2022). Current and future burden of breast cancer: Global statistics for 2020 and 2040. Breast.

[3] Zhang L., Feizi N., Chi C., Hu P. (2018). Association Analysis of Somatic Copy Number Alteration Burden with Breast Cancer Survival. Front. Genet..

[4] Jiang G., Zhang S., Yazdanparast A., Li M., Pawar A.V., Liu Y., Inavolu S.M., Cheng L. (2016). Comprehensive comparison of molecular portraits between cell lines and tumors in breast cancer. BMC Genom..

[5] Neve R.M., Chin K., Fridlyand J., Yeh J., Baehner F.L., Fevr T., Clark L., Bayani N., Coppe J.P., Tong F. (2006). A collection of breast cancer cell lines for the study of functionally distinct cancer subtypes. Cancer Cell.

[6] Sinn P., Aulmann S., Wirtz R., Schott S., Marme F., Varga Z., Lebeau A., Kreipe H., Schneeweiss A. (2013). Multigene Assays for Classification, Prognosis, and Prediction in Breast Cancer: A Critical Review on the Background and Clinical Utility. Geburtshilfe Frauenheilkd..

[7] Perou C.M., Sorlie T., Eisen M.B., van de Rijn M., Jeffrey S.S., Rees C.A., Pollack J.R., Ross D.T., Johnsen H., Akslen L.A. (2000). Molecular portraits of human breast tumours. Nature.

[8] Yersal O., Barutca S. (2014). Biological subtypes of breast cancer: Prognostic and therapeutic implications. World J. Clin. Oncol..

[9] Prat A., Perou C.M. (2011). Deconstructing the molecular portraits of breast cancer. Mol. Oncol..

[10] Weigelt B., Baehner F.L., Reis-Filho J.S. (2010). The contribution of gene expression profiling to breast cancer classification, prognostication and prediction: A retrospective of the last decade. J. Pathol..

[11] Prat A., Parker J.S., Karginova O., Fan C., Livasy C., Herschkowitz J.I., He X., Perou C.M. (2010). Phenotypic and molecular characterization of the claudin-low intrinsic subtype of breast cancer. Breast Cancer Res..

[12] Luís C., Guerra-Carvalho B., Braga P.C., Guedes C., Patrício E., Alves M.G., Fernandes R., Soares R. (2023). The Influence of Adipocyte Secretome on Selected Metabolic Fingerprints of Breast Cancer Cell Lines Representing the Four Major Breast Cancer Subtypes. Cells.

[13] Dai X., Cheng H., Bai Z., Li J. (2017). Breast Cancer Cell Line Classification and Its Relevance with Breast Tumor Subtyping. J. Cancer.

[14] Masci D., Naro C., Puxeddu M., Urbani A., Sette C., La Regina G., Silvestri R. (2023). Recent Advances in Drug Discovery for Triple-Negative Breast Cancer Treatment. Molecules.

[15] Limsakul P., Choochuen P., Jungrungrueang T., Charupanit K. (2024). Prognostic Markers in Tyrosine Kinases Specific to Basal-like 2 Subtype of Triple-Negative Breast Cancer. Int. J. Mol. Sci..

[16] Chapdelaine A.G., Sun G. (2023). Challenges and Opportunities in Developing Targeted Therapies for Triple Negative Breast Cancer. Biomolecules.

[17] O’Neill A.C., Alessandrino F., Tirumani S.H., Ramaiya N.H. (2018). Hallmarks of Cancer in the Reading Room: A Guide for Radiologists. Am. J. Roentgenol..

[18] Hanahan D. (2022). Hallmarks of Cancer: New Dimensions. Cancer Discov..

[19] Harrington J.J., Lieber M.R. (1994). The characterization of a mammalian DNA structure-specific endonuclease. EMBO J..

[20] Zheng L., Dai H., Zhou M., Li M., Singh P., Qiu J., Tsark W., Huang Q., Kernstine K., Zhang X. (2007). Fen1 mutations result in autoimmunity, chronic inflammation and cancers. Nat. Med..

[21] Zheng L., Jia J., Finger L.D., Guo Z., Zer C., Shen B. (2011). Functional regulation of FEN1 nuclease and its link to cancer. Nucleic Acids Res..

[22] Balakrishnan L., Bambara R.A. (2013). Flap endonuclease 1. Annu. Rev. Biochem..

[23] Harrington J.J., Lieber M.R. (1994). Functional domains within FEN-1 and RAD2 define a family of structure-specific endonucleases: Implications for nucleotide excision repair. Genes Dev..

[24] Zheng L., Zhou M., Chai Q., Parrish J., Xue D., Patrick S.M., Turchi J.J., Yannone S.M., Chen D., Shen B. (2005). Novel function of the flap endonuclease 1 complex in processing stalled DNA replication forks. EMBO Rep..

[25] Singh P., Yang M., Dai H., Yu D., Huang Q., Tan W., Kernstine K.H., Lin D., Shen B. (2008). Overexpression and Hypomethylation of Flap Endonuclease 1 Gene in Breast and Other Cancers. Mol. Cancer Res..

[26] Nolan J.P., Shen B., Park M.S., Sklar L.A. (1996). Kinetic analysis of human flap endonuclease-1 by flow cytometry. Biochemistry.

[27] Guo Z., Qian L., Liu R., Dai H., Zhou M., Zheng L., Shen B. (2008). Nucleolar localization and dynamic roles of flap endonuclease 1 in ribosomal DNA replication and damage repair. Mol. Cell. Biol..

[28] Alhmoud J.F., Woolley J.F., Al Moustafa A.E., Malki M.I. (2020). DNA Damage/Repair Management in Cancers. Cancers.

[29] Kinner A., Wu W., Staudt C., Iliakis G. (2008). Gamma-H2AX in recognition and signaling of DNA double-strand breaks in the context of chromatin. Nucleic Acids Res..

[30] Wu X., Wilson T.E., Lieber M.R. (1999). A role for FEN-1 in nonhomologous DNA end joining: The order of strand annealing and nucleolytic processing events. Proc. Natl. Acad. Sci. USA.

[31] Qiu J., Li X., Frank G., Shen B. (2001). Cell cycle-dependent and DNA damage-inducible nuclear localization of FEN-1 nuclease is consistent with its dual functions in DNA replication and repair. J. Biol. Chem..

[32] Ranalli T.A., Tom S., Bambara R.A. (2002). AP endonuclease 1 coordinates flap endonuclease 1 and DNA ligase I activity in long patch base excision repair. J. Biol. Chem..

[33] Krokan H.E., Standal R., Slupphaug G. (1997). DNA glycosylases in the base excision repair of DNA. Biochem. J..

[34] Robertson A.B., Klungland A., Rognes T., Leiros I. (2009). DNA repair in mammalian cells: Base excision repair: The long and short of it. Cell Mol. Life Sci..

[35] Prasad R., Singhal R.K., Srivastava D.K., Molina J.T., Tomkinson A.E., Wilson S.H. (1996). Specific interaction of DNA polymerase beta and DNA ligase I in a multiprotein base excision repair complex from bovine testis. J. Biol. Chem..

[36] Kikuchi K., Taniguchi Y., Hatanaka A., Sonoda E., Hochegger H., Adachi N., Matsuzaki Y., Koyama H., van Gent D.C., Jasin M. (2005). Fen-1 facilitates homologous recombination by removing divergent sequences at DNA break ends. Mol. Cell. Biol..

[37] Mengwasser K.E., Adeyemi R.O., Leng Y., Choi M.Y., Clairmont C., D’Andrea A.D., Elledge S.J. (2019). Genetic Screens Reveal FEN1 and APEX2 as BRCA2 Synthetic Lethal Targets. Mol. Cell.

[38] Becker J.R., Gallo D., Leung W., Croissant T., Thu Y.M., Nguyen H.D., Starr T.K., Brown G.W., Bielinsky A.-K. (2018). Flap endonuclease overexpression drives genome instability and DNA damage hypersensitivity in a PCNA-dependent manner. Nucleic Acids Res..

[39] Abdel-Fatah T.M., Russell R., Albarakati N., Maloney D.J., Dorjsuren D., Rueda O.M., Moseley P., Mohan V., Sun H., Abbotts R. (2014). Genomic and protein expression analysis reveals flap endonuclease 1 (FEN1) as a key biomarker in breast and ovarian cancer. Mol. Oncol..

[40] Xie Y., Dong B., Sun Z., Feng Y., Zhao W., Li K., Liu K., Cao J., Zhu C. (2023). FEN1 promotes cancer progression of cholangiocarcinoma by regulating the Wnt/β-catenin signaling pathway. Dig. Liver Dis..

[41] Wang K., Xie C., Chen D. (2014). Flap endonuclease 1 is a promising candidate biomarker in gastric cancer and is involved in cell proliferation and apoptosis. Int. J. Mol. Med..

[42] He L., Luo L., Zhu H., Yang H., Zhang Y., Wu H., Sun H., Jiang F., Kathera C.S., Liu L. (2017). FEN1 promotes tumor progression and confers cisplatin resistance in non-small-cell lung cancer. Mol. Oncol..

[43] Li J.L., Wang J.P., Chang H., Deng S.M., Du J.H., Wang X.X., Hu H.J., Li D.Y., Xu X.B., Guo W.Q. (2019). FEN1 inhibitor increases sensitivity of radiotherapy in cervical cancer cells. Cancer Med..

[44] Guo M., Wang S.M. (2021). Genome Instability-Derived Genes Are Novel Prognostic Biomarkers for Triple-Negative Breast Cancer. Front. Cell Dev. Biol..

[45] Wang J., Zhou L., Li Z., Zhang T., Liu W., Liu Z., Yuan Y.-C., Su F., Xu L., Wang Y. (2015). YY1 suppresses FEN1 over-expression and drug resistance in breast cancer. BMC Cancer.

[46] Xu L., Qu J.L., Song N., Zhang L.Y., Zeng X., Che X.F., Hou K.Z., Shi S., Feng Z.Y., Qu X.J. (2020). Biological and clinical significance of flap endonuclease-1 in triple-negative breast cancer: Support of metastasis and a poor prognosis. Oncol. Rep..

[47] Xu L., Shen J.M., Qu J.L., Song N., Che X.F., Hou K.Z., Shi J., Zhao L., Shi S., Liu Y.P. (2021). FEN1 is a prognostic biomarker for ER+ breast cancer and associated with tamoxifen resistance through the ERalpha/cyclin D1/Rb axis. Ann. Transl. Med..

[48] Bian S., Ni W., Zhu M., Zhang X., Qiang Y., Zhang J., Ni Z., Shen Y., Qiu S., Song Q. (2022). Flap endonuclease 1 Facilitated Hepatocellular Carcinoma Progression by Enhancing USP7/MDM2-mediated P53 Inactivation. Int. J. Biol. Sci..

[49] Sporikova Z., Koudelakova V., Trojanec R., Hajduch M. (2018). Genetic Markers in Triple-Negative Breast Cancer. Clin. Breast Cancer.

[50] Bartha Á., Győrffy B. (2021). TNMplot.com: A Web Tool for the Comparison of Gene Expression in Normal, Tumor and Metastatic Tissues. Int. J. Mol. Sci..

[51] Daukste L., Basse B., Baguley B.C., Wall D.J.N. (2012). Mathematical Determination of Cell Population Doubling Times for Multiple Cell Lines. Bull. Math. Biol..

[52] Győrffy B. (2023). Discovery and ranking of the most robust prognostic biomarkers in serous ovarian cancer. Geroscience.

[53] Győrffy B. (2021). Survival analysis across the entire transcriptome identifies biomarkers with the highest prognostic power in breast cancer. Comput. Struct. Biotechnol. J..

[54] Kazak L., Reyes A., He J., Wood S.R., Brea-Calvo G., Holen T.T., Holt I.J. (2013). A cryptic targeting signal creates a mitochondrial FEN1 isoform with tailed R-Loop binding properties. PLoS ONE.

[55] Guo E., Ishii Y., Mueller J., Srivatsan A., Gahman T., Putnam C.D., Wang J.Y.J., Kolodner R.D. (2020). FEN1 endonuclease as a therapeutic target for human cancers with defects in homologous recombination. Proc. Natl. Acad. Sci. USA.

[56] Lewanski C.R., Gullick W.J. (2001). Radiotherapy and cellular signalling. Lancet Oncol..

[57] Frey B., Borgmann K., Jost T., Greve B., Oertel M., Micke O., Eckert F. (2023). DNA as the main target in radiotherapy—A historical overview from first isolation to anti-tumour immune response. Strahlenther. Onkol..

[58] Redon C.E., Dickey J.S., Bonner W.M., Sedelnikova O.A. (2009). γ-H2AX as a biomarker of DNA damage induced by ionizing radiation in human peripheral blood lymphocytes and artificial skin. Adv. Space Res..

[59] Rothkamm K., Löbrich M. (2003). Evidence for a lack of DNA double-strand break repair in human cells exposed to very low x-ray doses. Proc. Natl. Acad. Sci. USA.

[60] Okamoto A., Utani K.-i., Shimizu N. (2011). DNA replication occurs in all lamina positive micronuclei, but never in lamina negative micronuclei. Mutagenesis.

[61] Bonassi S., El-Zein R., Bolognesi C., Fenech M. (2011). Micronuclei frequency in peripheral blood lymphocytes and cancer risk: Evidence from human studies. Mutagenesis.

[62] Gisselsson D., Björk J., Höglund M., Mertens F., Dal Cin P., Akerman M., Mandahl N. (2001). Abnormal nuclear shape in solid tumors reflects mitotic instability. Am. J. Pathol..

[63] Terradas M., Martín M., Genescà A. (2016). Impaired nuclear functions in micronuclei results in genome instability and chromothripsis. Arch. Toxicol..

[64] Cardinale F., Bruzzi P., Bolognesi C. (2012). Role of micronucleus test in predicting breast cancer susceptibility: A systematic review and meta-analysis. Br. J. Cancer.

[65] Luzhna L., Kathiria P., Kovalchuk O. (2013). Micronuclei in genotoxicity assessment: From genetics to epigenetics and beyond. Front. Genet..

[66] Badmus J.A., Ekpo O.E., Hussein A.A., Meyer M., Hiss D.C. (2019). Cytotoxic and cell cycle arrest properties of two steroidal alkaloids isolated from Holarrhena floribunda (G. Don) T. Durand & Schinz leaves. BMC Complement. Altern. Med..

[67] Lu W.-J., Peng W., Sun Q.-Q., Li Y.-H., Chen B., Yu L.-T., Xu Y.-Z., Wang S.-Y., Zhao Y.-L. (2018). #2714, a novel active inhibitor with potent G2/M phase arrest and antitumor efficacy in preclinical models. Cell Death Discov..

[68] Zhang M., Qu J., Gao Z., Qi Q., Yin H., Zhu L., Wu Y., Liu W., Yang J., Huang X. (2021). Timosaponin AIII Induces G2/M Arrest and Apoptosis in Breast Cancer by Activating the ATM/Chk2 and p38 MAPK Signaling Pathways. Front. Pharmacol..

[69] Krempler A., Deckbar D., Jeggo P.A., Löbrich M. (2007). An imperfect G2M checkpoint contributes to chromosome instability following irradiation of S and G2 phase cells. Cell Cycle.

[70] Anastasov N., Höfig I., Vasconcellos I.G., Rappl K., Braselmann H., Ludyga N., Auer G., Aubele M., Atkinson M.J. (2012). Radiation resistance due to high expression of miR-21 and G2/M checkpoint arrest in breast cancer cells. Radiat. Oncol..

[71] Sinclair W.K. (1968). Cyclic X-Ray Responses in Mammalian Cells in Vitro. Radiat. Res..

[72] White T.L., Deshpande N., Kumar V., Gauthier A.G., Jurkunas U.V. (2021). Cell cycle re-entry and arrest in G2/M phase induces senescence and fibrosis in Fuchs Endothelial Corneal Dystrophy. Free Radic. Biol. Med..

[73] Kim D., Lee J., Han J., Lim J., Lim E.K., Kim E. (2023). A highly specific and flexible detection assay using collaborated actions of DNA-processing enzymes for identifying multiple gene expression signatures in breast cancer. Analyst.

[74] Viktorsson K., Rieckmann T., Fleischmann M., Diefenhardt M., Hehlgans S., Rödel F. (2023). Advances in molecular targeted therapies to increase efficacy of (chemo)radiation therapy. Strahlenther. Onkol..

[75] Qi L., Zhou B., Chen J., Hu W., Bai R., Ye C., Weng X., Zheng S. (2019). Significant prognostic values of differentially expressed-aberrantly methylated hub genes in breast cancer. J. Cancer.

[76] Lewis C.W., Golsteyn R.M. (2016). Cancer cells that survive checkpoint adaptation contain micronuclei that harbor damaged DNA. Cell Cycle.

[77] Tumey L.N., Bom D., Huck B., Gleason E., Wang J., Silver D., Brunden K., Boozer S., Rundlett S., Sherf B. (2005). The identification and optimization of a N-hydroxy urea series of flap endonuclease 1 inhibitors. Bioorg. Med. Chem. Lett..

[78] Elstrodt F., Hollestelle A., Nagel J.H.A., Gorin M., Wasielewski M., van den Ouweland A., Merajver S.D., Ethier S.P., Schutte M. (2006). BRCA1 Mutation Analysis of 41 Human Breast Cancer Cell Lines Reveals Three New Deleterious Mutants. Cancer Res..

[79] Fenech M. (2000). The in vitro micronucleus technique. Mutat. Res..

[80] Fenech M., Kirsch-Volders M., Natarajan A.T., Surralles J., Crott J.W., Parry J., Norppa H., Eastmond D.A., Tucker J.D., Thomas P. (2011). Molecular mechanisms of micronucleus, nucleoplasmic bridge and nuclear bud formation in mammalian and human cells. Mutagenesis.

[81] Mackenzie K.J., Carroll P., Martin C.-A., Murina O., Fluteau A., Simpson D.J., Olova N., Sutcliffe H., Rainger J.K., Leitch A. (2017). cGAS surveillance of micronuclei links genome instability to innate immunity. Nature.

[82] Braselmann H., Michna A., Heß J., Unger K. (2015). CFAssay: Statistical analysis of the colony formation assay. Radiat. Oncol..

[83] Franken N.A.P., Rodermond H.M., Stap J., Haveman J., van Bree C. (2006). Clonogenic assay of cells in vitro. Nat. Protoc..

[84] Cahu J., Sola B. (2013). A sensitive method to quantify senescent cancer cells. J. Vis. Exp..

[85] Debacq-Chainiaux F., Erusalimsky J.D., Campisi J., Toussaint O. (2009). Protocols to detect senescence-associated beta-galactosidase (SA-βgal) activity, a biomarker of senescent cells in culture and in vivo. Nat. Protoc..

[86] Bertolo A., Baur M., Guerrero J., Pötzel T., Stoyanov J. (2019). Autofluorescence is a Reliable in vitro Marker of Cellular Senescence in Human Mesenchymal Stromal Cells. Sci. Rep..

[87] Jost T., Heinzerling L., Fietkau R., Hecht M., Distel L.V. (2021). Palbociclib Induces Senescence in Melanoma and Breast Cancer Cells and Leads to Additive Growth Arrest in Combination with Irradiation. Front. Oncol..

