# Supplementary material for: FEN1 Inhibition as a Potential Novel Targeted Therapy against Breast Cancer and the Prognostic Relevance of FEN1
Source: Int J Mol Sci. 2024 Feb 9;25(4):2110. doi: 10.3390/ijms25042110 (PMC10889347; doi:10.3390/ijms25042110)
Supplement: Supplementary file 1 [file ijms-25-02110-s001.zip › ijms-2863197-supplementary.pdf]

FEN1 Inhibition as a Potential Novel Targeted Therapy Against Breast Cancer and the Prognostic Relevance of FEN1

Johanna Berfelde [1], Laura S. Hildebrand [1,2], Lukas Kuhlmann [1,2], Rainer Fietkau [1,2], and Luitpold V. Distel [1,2,*]

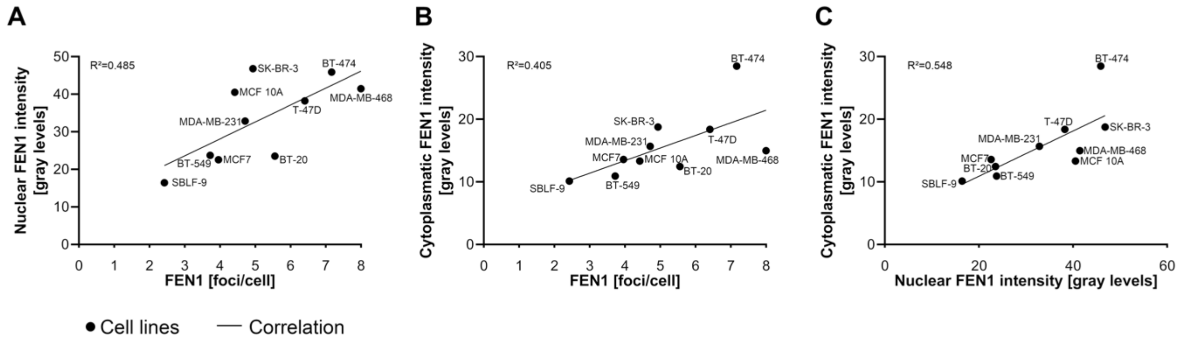

Figure S1. Association between nuclear FEN1 foci number and FEN1 intensity of untreated cells. The linear regression analysis was performed with all cell lines and is displayed as line. The values represent the mean value of at least four independent experiments. (**A**) Linear regression analysis of FEN1 foci and nuclear FEN1 intensity. (**B**) Linear regression analysis of FEN1 foci and cytoplasmatic FEN1 intensity. (**C**) Linear regression analysis of nuclear FEN1 intensity and cytoplasmatic FEN1 intensity.

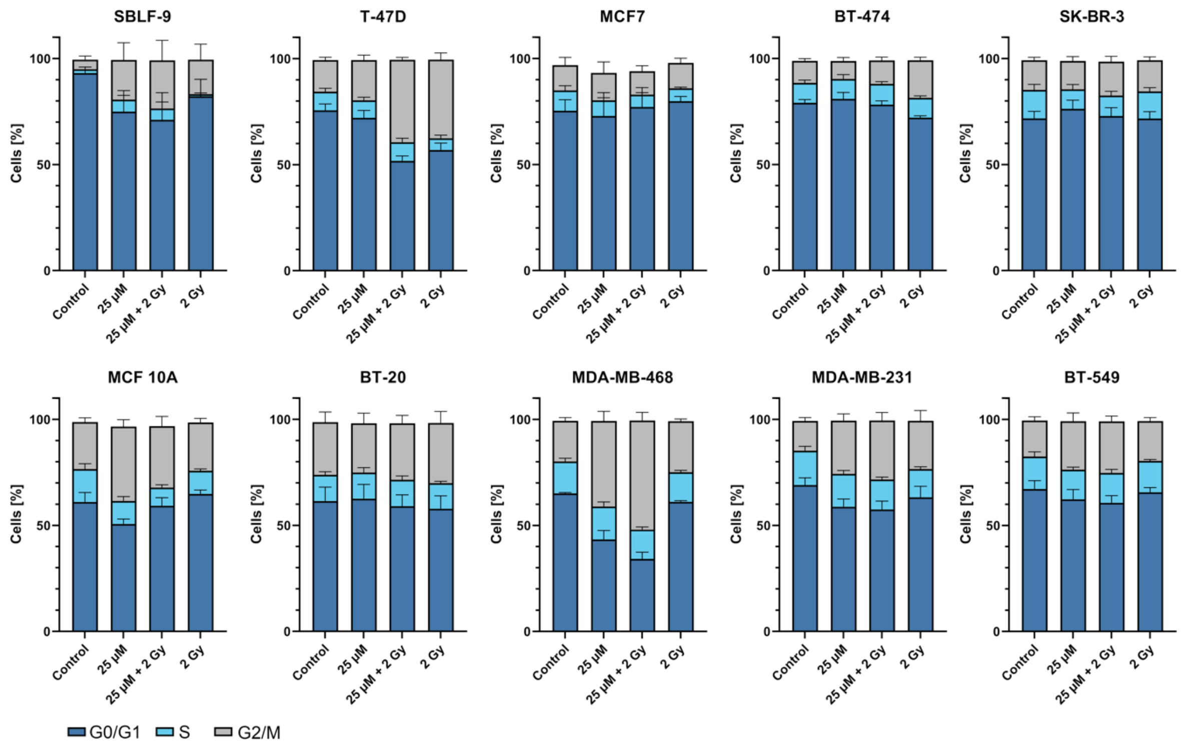

Figure S2. Representation of the relative distribution of cells in the cell cycle 2 days after treatment with FEN1-IN-4 with or without IR. Breast cancer cell lines are grouped according to hormone receptor status, mammary epithelial cells and healthy fibroblasts serve as controls. Each experiment was repeated independently at least four times, the resulting mean value and the standard deviation are shown.

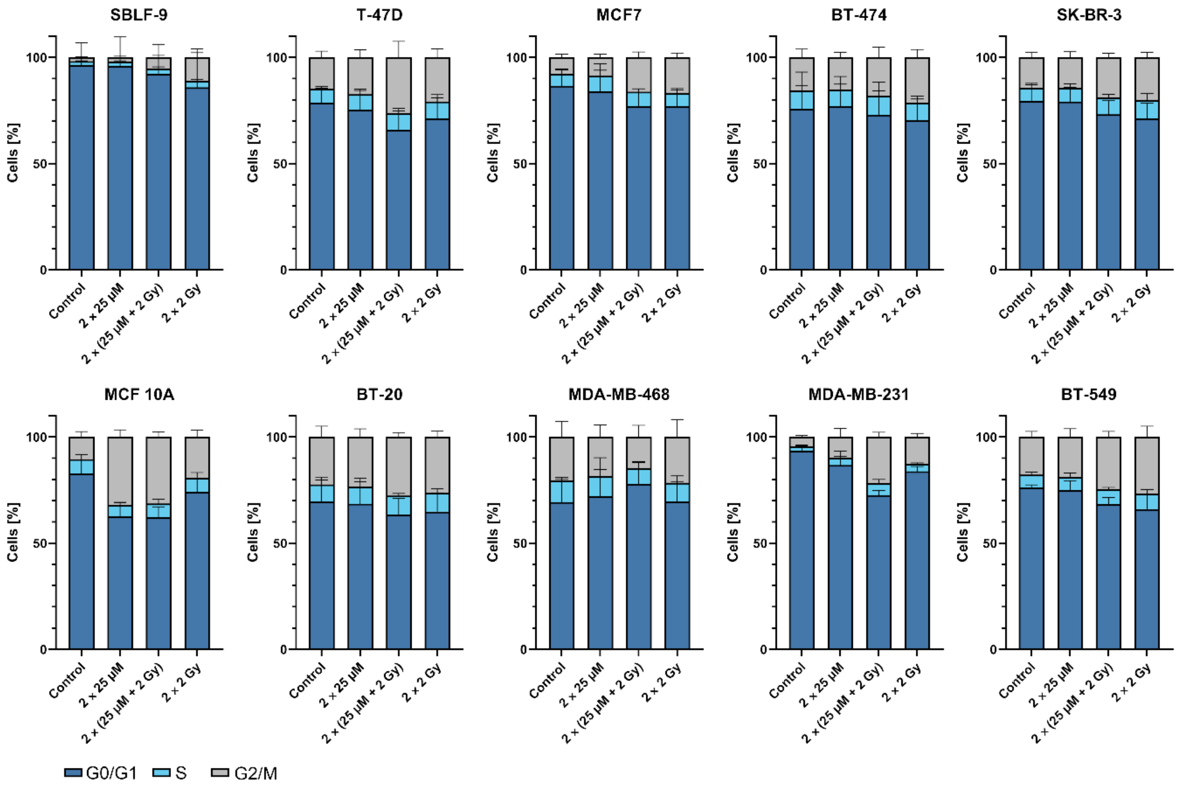

Figure S3. Percentage distribution of cells in the cell cycle phases after double therapy with FEN1-IN-4 with or without IR and a total incubation time of 10 days. The mean value and the standard deviation of at least four independent experiments are displayed. Breast cancer cell lines are grouped according to hormone receptor status next to mammary epithelial cells (MCF 10A) and healthy fibroblasts (SBLF-9).

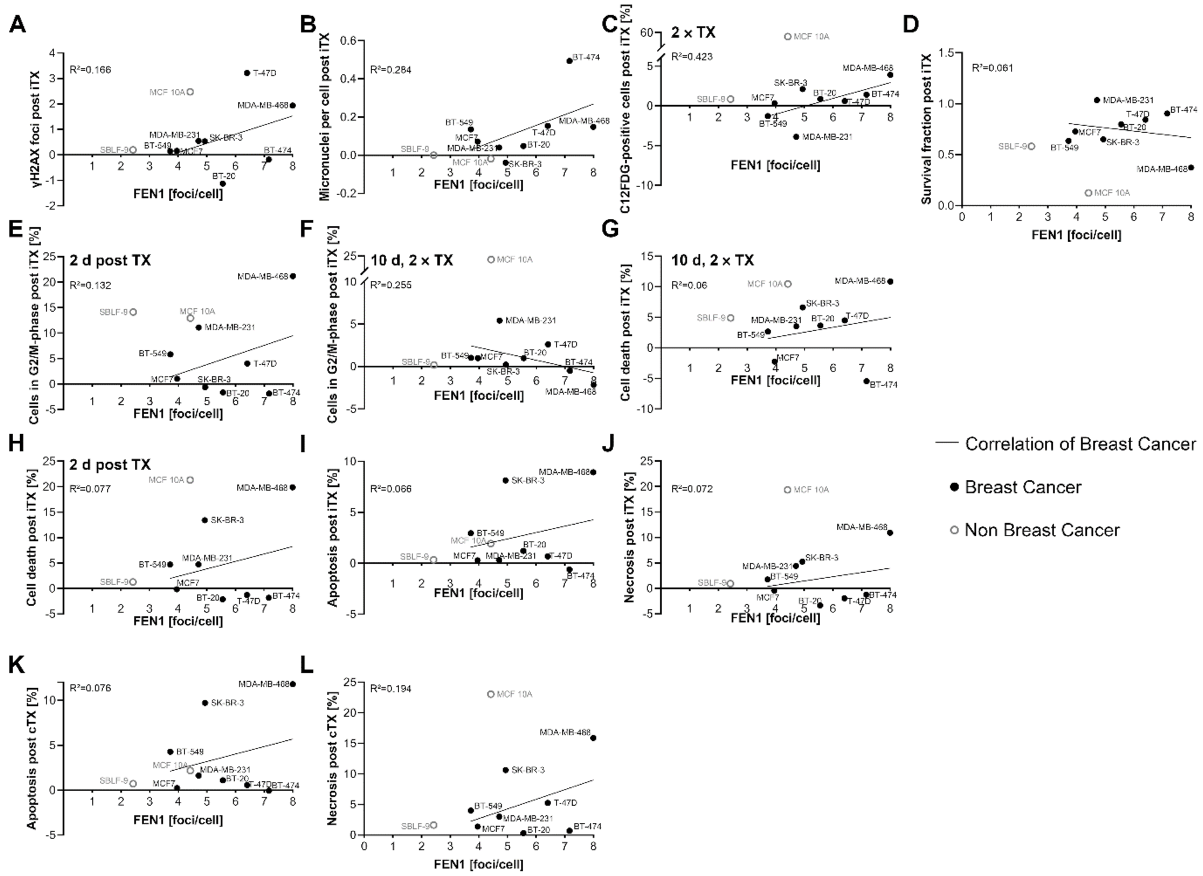

Figure S4. Relationship between FEN1 foci quantity and response to therapy. Only tumor cells were included in the calculation, non-malignant cell lines are nevertheless shown in a different color for comparison. The line represents linear regression, and the values represent the mean value of at least four independent experiments. The respective baseline values of the control were subtracted from those of the combination or inhibitor therapy. iTX, FEN1-IN-4 monotherapy. Association between FEN1 foci quantity of untreated cells and effects of FEN1-IN-4 monotherapy (**A-J**) or FEN1-IN-4 combined with IR (**K-L**).
